# *Helicobacter pylori*-infected C57BL/6 mice with different gastrointestinal microbiota have contrasting gastric pathology, microbial and host immune responses

**DOI:** 10.1038/s41598-018-25927-2

**Published:** 2018-05-22

**Authors:** Zhongming Ge, Alexander Sheh, Yan Feng, Sureshkumar Muthupalani, Lili Ge, Chuanwu Wang, Susanna Kurnick, Anthony Mannion, Mark T. Whary, James G. Fox

**Affiliations:** Division of Comparative Medicine, the Massachusetts Institute of Technology, Cambridge, MA 02139 USA

## Abstract

C57BL/6 (B6) mice from Taconic Sciences (Tac) and the Jackson Laboratory (Jax) were infected with *H*. *pylori* PMSS1 (Hp) for 16 week; there was no significant difference in the gastric histologic activity index between Hp infected Tac and Jax B6. However, the degree of gastric mucous metaplasia and Th1-associated IgG2c levels in response to Hp infection were increased in Tac mice over Jax mice, whereas the colonization levels of gastric Hp were higher by 8-fold in Jax B6 compared with Tac B6. Additionally, mRNA expression of gastric *Il-1β*, *Il-17A* and *RegIIIγ* were significantly lower in the infected Tac compared to the infected Jax mice. There were significant differences in the microbial community structures in stomach, colon, and feces between Jax and Tac B6 females. Differences in gastric microbial communities between Jax and Tac B6 females are predicted to affect the metagenome. Moreover, Hp infection perturbed the microbial community structures in the stomach, colon and feces of Jax mice, but only altered the colonic microbial composition of Tac mice. Our data indicate that the GI microbiome of Tac B6 mice is compositionally distinct from Jax B6 mice, which likely resulted in different pathological, immunological, and microbial responses to Hp infection.

## Introduction

*Helicobacter pylori* infection can cause chronic active gastritis, peptic ulcers, and is a major risk factor for the development of gastric adenocarcinoma^1,2^. Approximately 50% of the world population is infected with this pathogen, ~10% of which develop gastric or duodenal ulcer disease and ~1–3% of *H*. *pylori*-positive patients are diagnosed with gastric cancer^3^. Pathogenicity of *H*. *pylori* infection is influenced by multiple factors, including virulence properties of *H*. *pylori* strains, host factors, and environmental conditions^4–6^. Cumulative data on gastrointestinal microbiomes have revealed that there are significant differences in the gastric microflora between *H*. *pylori*-positive and –negative patients or between *H*. *pylori*-positive patients with and without gastric cancer^7–9^. *H*. *pylori* infection perturbed the gastric microbial compositions of specific pathogen-free INS-GAS mice and in a second study the presence of the restricted microbiota consisting of 3 ASF strains accelerated progression of *H*. *pylori*-induced gastric pathology in INS-GAS mice^10,11^. Additionally, *H*. *pylori* induced gastric pathology in C57BL/6 can be attenuated by co-infection with *H*. *bilis* or *H*. *muridarum*, or be promoted by *H*. *hepaticus* co-infection, all of which are enterohepatic helicobacters primarily colonizing the large intestine^12,13^. A recent study demonstrated that *H*. *pylori* infection in C57BL/6 mice altered both gastric and the intestinal microbial composition and also modulated host immune responses in both the stomach and lung^14^. However, the effects of different gastrointestinal (GI) microbial compositions on disease development in the individuals with a closely related genetic background are poorly understood. This variable would be difficult to be dissected in a human population due to variability of multiple factors such as genetic heterogeneity, dietary habits and living conditions.

Th1-biased C57BL/6 mice have been used for delineating the mechanisms of inflammation driven gastric carcinogenesis induced by *H*. *pylori* infection^15^. Distinct ileal microbiota were observed in C57BL/6 mice from the Jackson laboratory (Jax) and Taconic Bioscience (Tac)^16^. Most notably, segmented filamentous bacteria (SFB), a group of spore-forming gram-positive bacteria, are present in Tac mice but absent from Jax mice. SFB promote proinflammatory Th17 cell responses and also play an important role in inducing mucosal IgA responses^16–21^. Given the role of the microbiome, and in particular SFB, in shaping host immune responses, we hypothesized that *H*. *pylori* infection induces pathological effects and immune responses differentially in C57BL/6 mice with different gastrointestinal microbiota, which could provide new insights into the effects of different GI microbiota on *H*. *pylori*-induced gastric pathology in humans. In this study, severity of gastric pathology, expression of inflammation-related mediators, the gastrointestinal (GI) microbiomes (stomach, colon, feces) and colonization dynamics of SFB in response to *H*. *pylori* infection in C57BL/6 mice from Jax and Tac were characterized and compared.

## Results

### *H*. *pylori*-infected Tac mice developed more severe gastric mucous metaplasia than the *H*. *pylori* infected Jax mice

**Figure 1:**
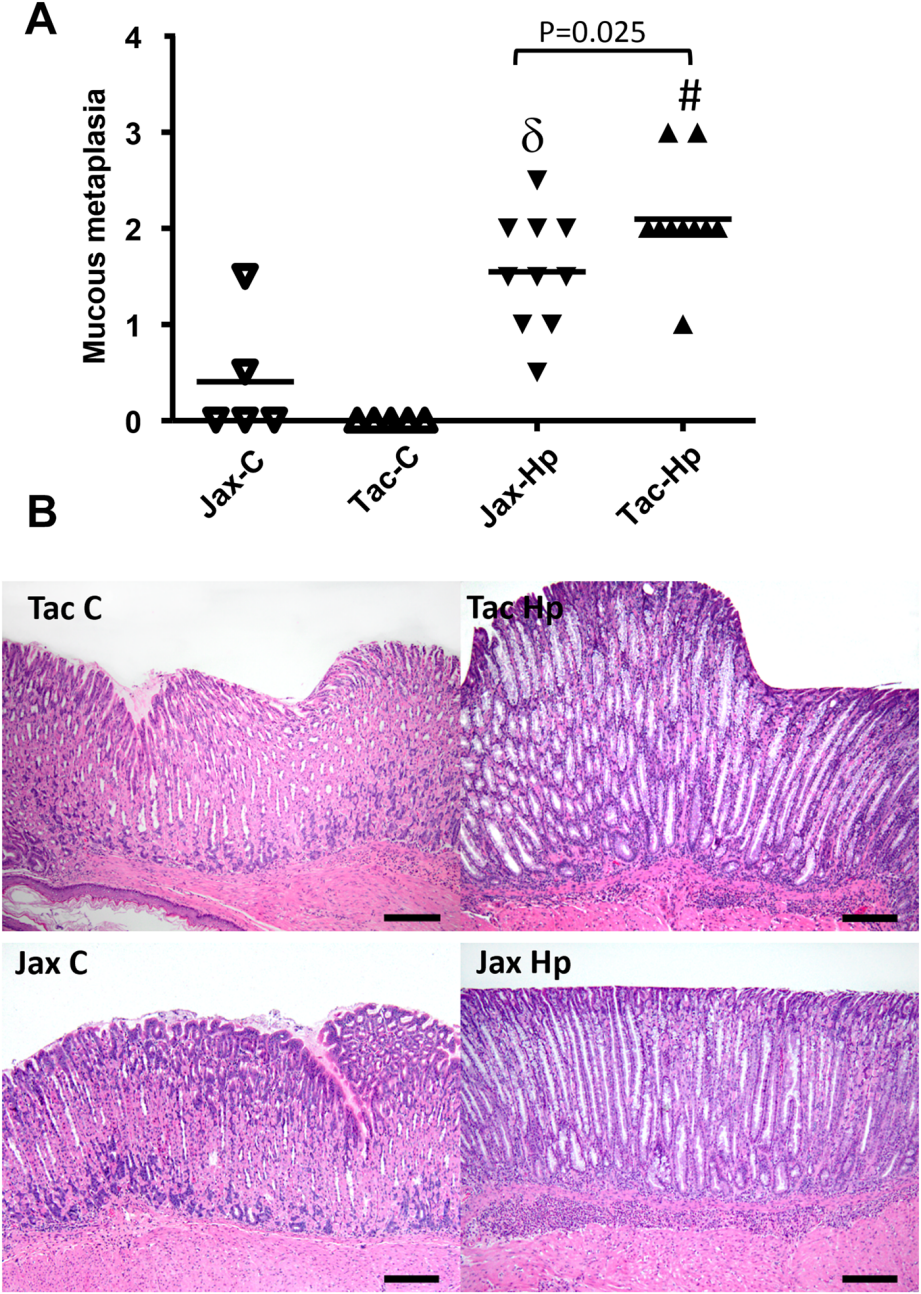
Severity of mucous metaplasia was increased in *H*. *pylori*-infected Tac B6 mice compared with their Jax counterparts. (**A**) Scores (ascending from 1 to 4) of gastric mucous metaplasia that was more severe in the infected Tac mice. (**B**) Representative images showing pathological features among the groups.

At 16 weeks post *H*. *pylori* inoculation (16 WPI), *H*. *pylori* infection induced significantly more severe gastric pathology in both Tac and Jax B6 mice compared to their sham controls (Fig. S1, P < 0.003). Severity of gastric pathology as represented by gastric histologic activity index (GHAI) was comparable between infected Tac and Jax mice (P = 0.68). There were no significant differences for categorical features including inflammation, epithelial defects, oxyntic atrophy, hyperplasia and dysplasia in the infected mice from between these 2 vendors (Fig. S1). However, *H*. *pylori* infected Tac mice developed significantly more severe gastric mucous metaplasia compared to infected Jax mice (Fig. 1A, P < 0.05). The scores of the mucous metaplasia in these infected Tac and Jax mice was also significantly higher compared with their sham controls (P < 0.02), indicating that this pathological entity was specifically induced by *H*. *pylori* infection (Fig. 1B).

**Figure 2:**
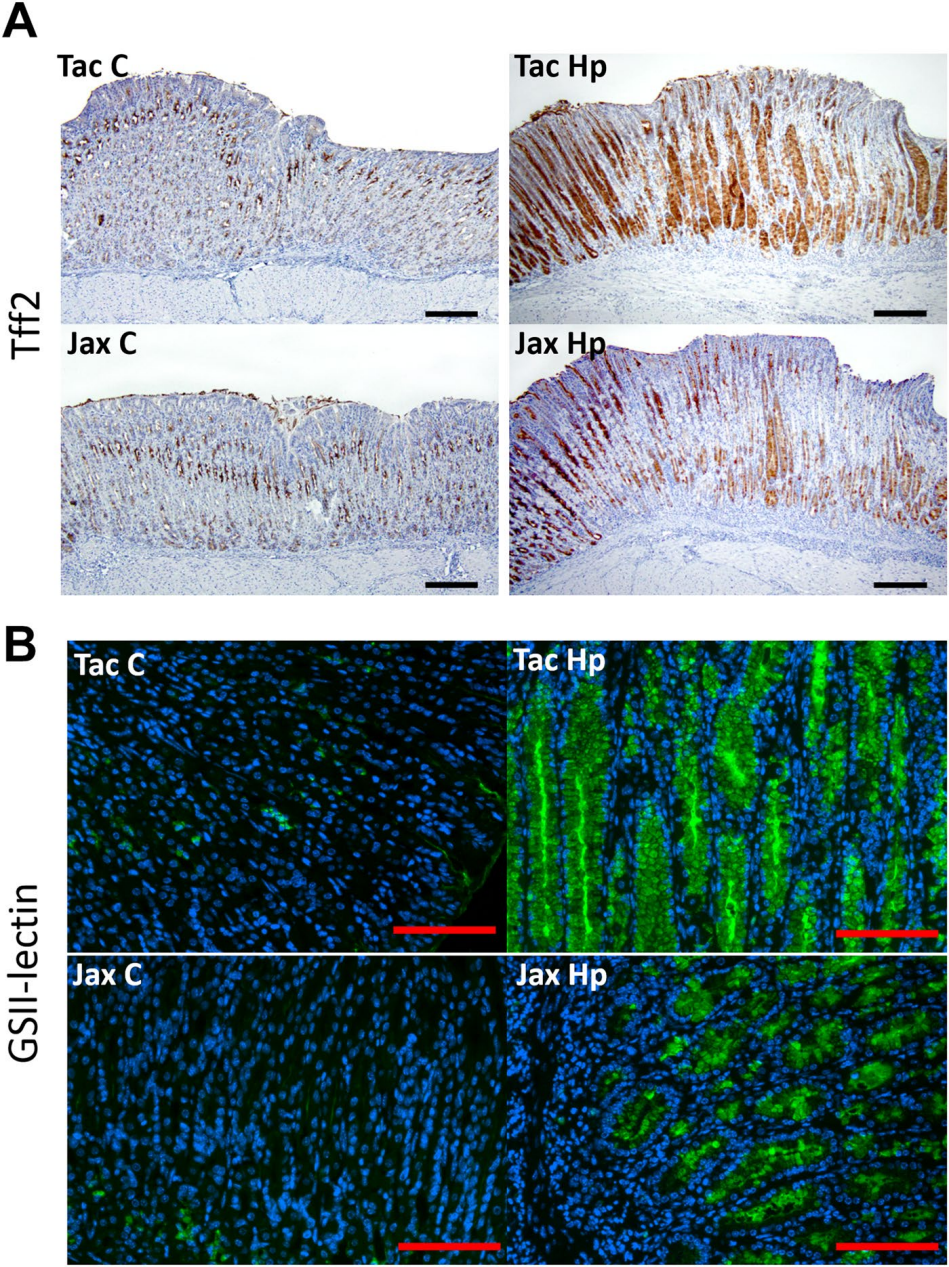
Expression of Tff2 and GSII was enhanced in the gastric corpus of the infected Tac B6 compared with their Jax counterparts. (**A**) Tff2 IHC and (**B**) GSII immunofluorescence.

To further characterize the origins of mucous metaplasia, we examined expression patterns of markers for mucous metaplasia (Tff2) and SPEM (Tff2, GSII-lectin) in the gastric tissues using immunohistochemistry^22–24^. There was very mild Tff2 expression in the gastric corpus of both Jax and Tac controls restricted to mucous neck cells and to select basal glands, whereas in *H*. *pylori* infected mice, there was an increased and *de novo* immunostaining of the hyperplastic and metaplastic glands and/or foveolar pits and in many foci spanning across their entire length of the mucosa (Fig. 2A). Additionally, expression of Tff2 was more pronounced in the stomach of the *H*. *pylori*-infected Tac mice compared to the infected Jax mice primarily within segments that had undergone mucosa metaplasia, thus correlating with the increased mucous metaplasia observed in infected Tac mice. Similarly, there was few GSII-positive glands noted in the stomach of the controls of both vendors (Fig. 2B). In contrast, expression of gastric GSII-lectin was significantly increased in all the infected mice compared with the uninfected controls. Further, there was elevated expression of the GSII-lectin in the infected Tac B6 compared with the *H*. *pylori*-infected Jax B6 (Fig. 2B).

### Colonization levels of gastric *H*. *pylori* were significantly higher in Jax mice compared to Tac mice

**Figure 3:**
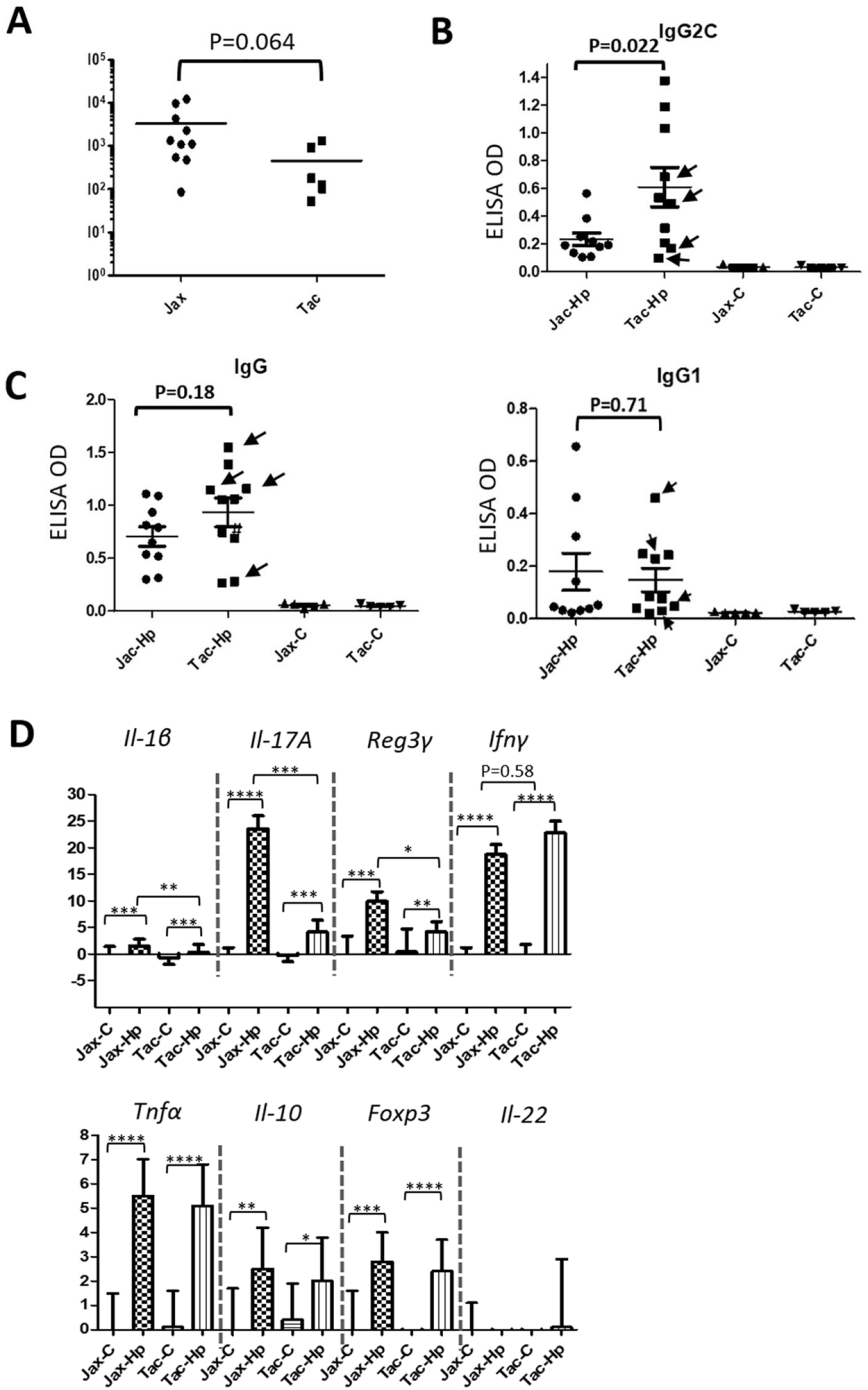
*H*. *pylori* colonization, serum IgG responses to *H*. *pylori* infection, and expression of select host genes were compared between Jax and Tac mice. (**A**) Gastric *H*. *pylori* colonization levels that was higher in Jax mice than Tac mice. The numbers on the y axis represent the copies of *H*. *pylori* genome expressed per µg murine DNA in the samples. (**B**) *H*. *pylori*-specific IgG2c levels measured with ELISA. (**C**) *H*. *pylori*-specific total IgG and Th2-associated IgG1 levels measured with ELISA. (**D**) mRNA levels of the target genes in stomach. All the mRNA levels were normalized to the expression of the housekeeping gene *Gapdh*. The *y* axes represent the mean fold changes (±standard deviations) of the mRNA levels in reference to uninfected Jax controls. P values: *<0.05. **<0.01, ***<0.001, ****<0.0001. Values indicated by arrows in B and C from *H*. *pylori*-infected Tac mice which were *H*. *pylori*-negative at 16 WPI.

Age-matched female C57BL/6 mice from Jax and Tac were infected with *H*. *pylori* PMSS1 for 16 weeks. All infected Jax mice were positive for *H*. *pylori*, whereas 4 of 10 infected Tac mice were negative for *H*. *pylori* (Fig. 3A). Additionally, the average colonization level of gastric *H*. *pylori* were higher by 8-fold in Jax mice (3317 ± 1345) compared to that in *H*. *pylori*-positive Tac mice (452 ± 219) without statistical significance (Fig. 3A, P = 0.064).

### Tac mice developed significantly stronger Th1-associated IgG2c response to *H*. *pylori* infection compared to Jax mice

Since colonization levels of gastric *H*. *pylori* were different between the infected Tac and Jax mice, we next examined IgG responses, a surrogate biomarker for *H*. *pylori* infection in *H*. *pylori*-infected mice and humans, to *H*. *pylori* infection (Fig. 3B,C). Tac mice produced higher levels of the Th1-associated IgG2c response to *H*. *pylori* compared to Jax mice (P < 0.03, Fig. 3B). However, there was no significant difference in both the total IgG and the Th2-associated IgG1 levels against *H*. *pylori* PMSS1 between Tac and Jax mice and (Fig. 3C). Notably, the levels of *H*. *pylori* specific serum IgG, IgG2c and IgG1 were comparable between *H*. *pylori*-negative (indicated by arrows in Fig. 3B,C) and *H*. *pylori*–positive mice in the *H*. *pylori*-infected Tac group.

### *H*. *pylori* infection induced higher expression of gastric *Il-1β*, *Il-17* and *RegIIIγ* in Jax mice compared to Tac mice

We previously showed that *H*. *pylori* infection elevated mRNA levels of gastric proinflammatory cytokines *Ifnγ*, *Tnfα*, *Il-1β* (Th1-type), *Il-17A* (Th17-type), *Il-22* (Th22-type) as well as anti-inflammatory mediators *Il-10* and *Foxp3* in C57BL/6 mice^12,13,25^. We therefore examined whether expression of these gastric cytokines and *RegIIIγ* (coding for an antimicrobial peptide) had differential responses to *H*. *pylori* infection between Jax and Tac B6 mice. The mRNA expression levels of all tested genes except for *Il-22* were significantly increased in the infected mice of both vendors compared to their sham controls (Fig. 3D, P < 0.05 or greater). No significant difference in mRNA expression of gastric *Il-22* between *H*. *pylori*-infected and control B6 mice at 16 WPI in this study was consistent with a previous report showing that *H*. *pylori*-induced elevation of gastric *Il-22* transcription in BALB/c and B6 mice peaked at 35 days after *H*. *pylori* infection and then was decreased to the same level as uninfected controls by 2 months after *H*. *pylori* infection^25^. Infected Jax mice produced significantly higher mRNA levels of gastric *Il-1β*, *Il-17A* and *RegIIIγ* compared to the infected Tac mice, whereas there was no significant differences in mRNA levels of gastric *Ifnγ*, *Tnfα*, *Il-10* and *Foxp3* (Fig. 3D). There were no significant differences in mRNA levels of these genes in the stomach between *H*. *pylori*-negative and –positive mice in the *H*. *pylori*-infected Tac group.

It has been documented that the presence of ileal SFB induces a robust Th17 response^16^. Thus, transcript levels of all genes examined on gastric tissues were also measured in the ileum of both infected and control mice. Transcript levels of ileal *Il-17A* and *Ifnγ* were significantly elevated in Tac mice compared to Jax mice (Fig. S2). The mRNA levels of ileal *Tnfα*, *Il-1β*, *Il-22*, *Il-10*, *Foxp3* and *RegIIIγ* were comparable between Tac and Jax mice regardless of infection status.

### *H*. *pylori* infection significantly increased colonization levels of SFB in the large intestine of Tac B6 mice

**Figure 4:**
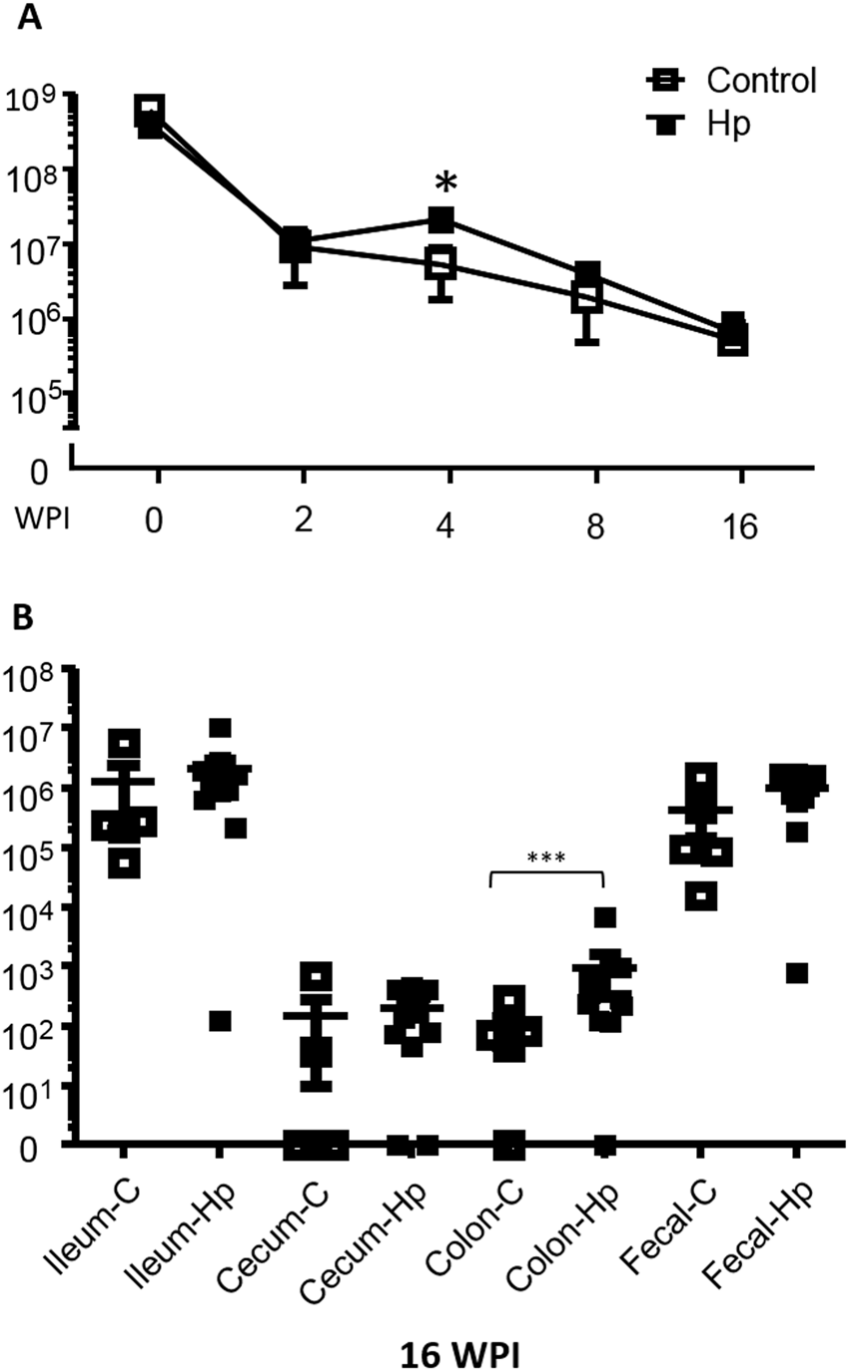
*H*. *pylori* infection increased prevalence rate and colonization levels of SFB in the cecum and colon of Tac mice, respectively. (**A**) Fecal SFB levels were significantly decreased 2 weeks post *H*. *pylori* infection and then gradually went lower form 4 to 16 WPI. (**B**) Colonization dynamics of SFB over ileum, cecum, colon and feces with and without *H*. *pylori* infection. The numbers on the y axis represent the SFB 16S rRNA gene copies of which are expressed per µg murine DNA in the corresponding samples. P values: *<0.05. **<0.01, ***<0.001.

By using SFB-specific qPCR^26^, we monitored fecal SFB levels over a time course in Jax and Tac mice and also measured colonization dynamics of SFB in the ileum, cecum and colon. All Jax mice were negative for SFB in these tissues analyzed. In contrast. SFB were present in the feces and ileum of all Tac mice, and select mice were positive for SFB in the colon and cecum (Fig. 4). After one week of housing Tac B6 mice at the MIT facility, fecal SFB levels were significantly decreased compared to levels upon arrival (Fig. 4A). Subsequently, SFB levels were gradually reduced from 2 to 16 WPI. *H*. *pylori* infection did not significantly alter fecal SFB levels except for 4 WPI when *H*. *pylori* infection significantly increased fecal SFB levels.

At 16 WPI, SFB primarily colonized the ileum, which is consistent with previous reports^16^. There was no significant difference in ileal SFB levels between *H*. *pylori* infected and control mice. However, *H*. *pylori* infection increased cecal SFB positivity (80% colonized) compared to the sham controls (40% colonized); there were significant higher levels of colonic SFB in the *H*. *pylori* infected mice compared to the sham controls (Fig. 4B, P < 0.03).

### Analysis of bacterial diversity and abundance in the stomach, colon and feces of Tac B6 and Jax B6 mice

Accumulating evidence has indicated alterations of the microbial community structures in the GI tract are associated with disease status in humans and experimental animal models^27–29^. The alpha diversity indices, Shannon and Observed species, were used to compare differences in vendor-associated microbiota in the stomach, colon and feces, of which gastric and colonic microbial community structures were mucosa-associated. The diversity of the gastric microbiota was similar between both vendors. However, alpha diversity indices demonstrated that the microbiota of Tac mice in the colon and the feces were significantly more diverse than in the colon and the feces of Jax mice, respectively (P values colon: 0.003 - Observed Species and 0.036 – Shannon. P values feces: both 0.003).

**Figure 5:**
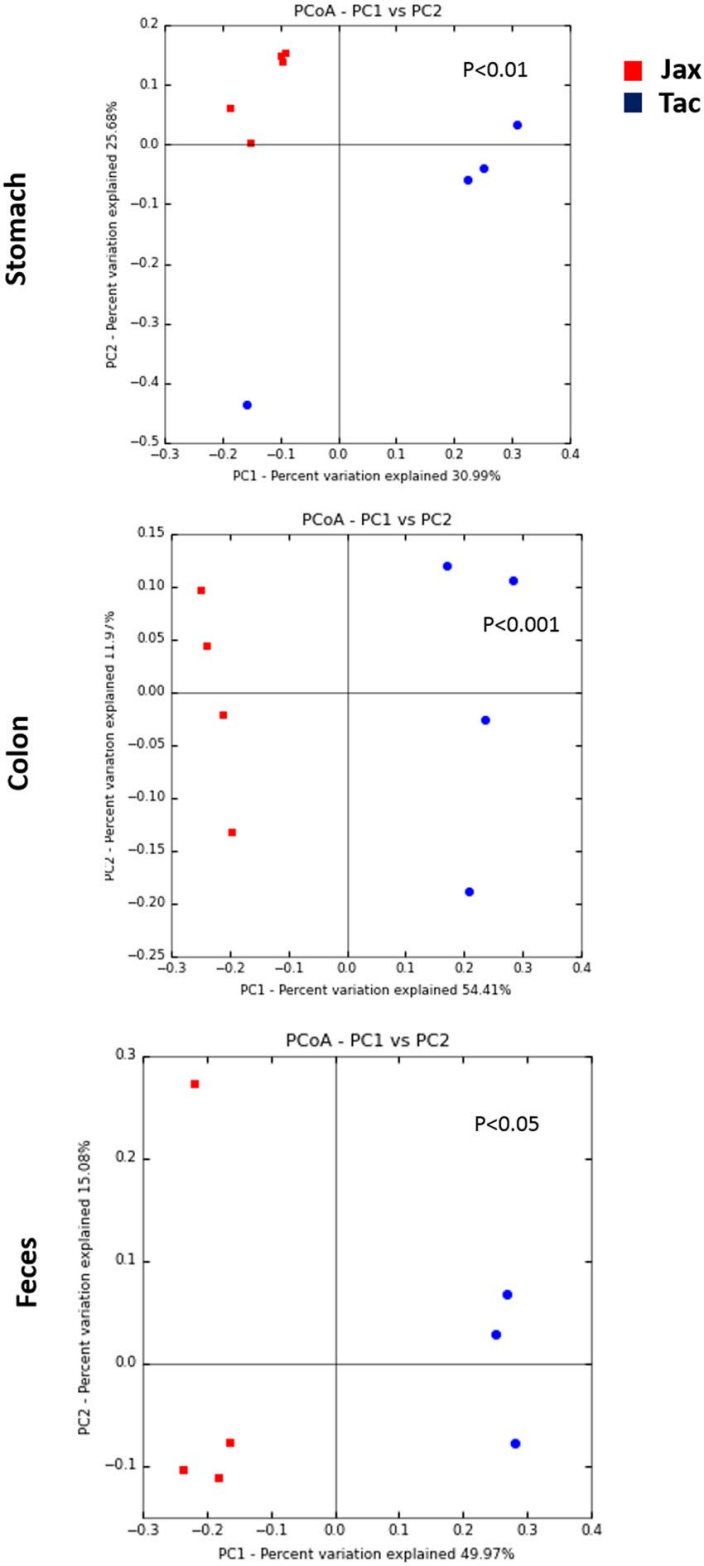
Comparison of gastric, colonic and fecal microbial community structures between Jax and Tac B6 female mice. Principal coordinates analysis (PCoA) (the unweighted UniFrac) was performed based on bacterial 16S rRNA gene sequences at an even sample depth (2743). Samples from Jax (orange) and Tac (red) mice are represented as colored circles. P values are calculated on the corresponding unweighted UniFrac distance matrix using ADONIS test and <0.05 is considered significant.

When beta diversity was analyzed using unweighted UniFrac, microbial flora from each sampled site were clustered predominantly by vendor (Fig. 5). PCoA analysis indicates that significant differences in the microbial community structures existed in the stomach (P < 0.01), colon (P < 0.001) and feces (P < 0.05) between the two vendors (Fig. 5). These results suggest that changes in the bacterial communities of the stomach, colon and feces between the vendors involved the loss or gain of specific bacterial genera.

Ten known phyla were identified in B6 mice from both vendors (Fig. S3A). The three most abundant phyla in each site was dependent on the source of the mice. For Jax mice, *Firmicutes*, *Proteobacteria* and *Bacteroidetes* were the most common in the stomach of both Jax (65.19%, 16.55% and 15.36%, respectively) and Tac (66.48%, 16.16%, and 14.82%, respectively) mice. Differences were observed in the colon, as Jax mice were predominantly colonized by *Firmicutes* (75.52%), *Bacteroidetes* (21.44%) and *Tenericutes* (1.88%), while Tac mice were colonized predominantly colonized by *Firmicutes* (92.07%), with smaller proportions of *Deferribacteres* (2.86%) and *Bacteroidetes* (2.53%). A larger shift was noted in the feces; Jax mice feces were composed mainly of *Bacteroidetes* (66.25%), followed by *Firmicutes* (31.63%) and *Tenericutes* (1.91%), whereas Tac mice had higher levels of *Firmicutes* (78.36%), with *Bacteroidetes* (17.91%) and *Tenericutes* (1.52%) rounding out the top three. Abundance of *Bacteroidetes* in the stomach, colon and feces of Jax mice was higher compared with Tac mice; the opposite was true for *Firmicutes* in Tac mice (Fig. S3A). In addition, phyla *Deferribacteres* and *Verrucomicrobia*, composed mainly of the genera *Mucispirillum* and *Akkermansia*, were only present in Tac mice. At the family level, there are more *Porphyromonadaceae* and *Rikenellaceae* in Tac mice and more S24-7 and *Turicibacteriaceae* in Jax mice when compared with the respective Tac counterparts (Fig. S3B). Statistical analysis on the relative abundance of bacterial groups at the family level was performed using LEfSe in uninfected control mice from both vendors as summarized in Table S1. In the stomach of control Tac mice, *Moraxellaceae*, *Rikenellaceae*, *Oxalobacteraceae*, *Deferribacteraceae*, and *Porphyromonadaceae* were enriched compared to Jax mice. In the stomachs of control Jax mice, an unassigned family from order RF39, *Bifidobacteriaceae*, bacteria from an unassigned family, *Coriobacteriaceae*, *Clostridiaceae*, *Turicibacteraceae*, and S24_7 were enriched compared to the Tac mice. In the colon, *Lachnospiraceae*, *Deferribacteraceae*, *Rikenellaceae*, *Verrucomicrobiaceae*, and *Porphyromonadaceae* were higher in control Tac mice, whereas *Turicibacteraceae*, bacteria from an unassigned family, *Bifidobacteriaceae*, and S24_7 were higher in the colon of control Jax mice. In feces, control Taconic mice had more *Ruminococcaceae*, *Rikenellaceae*, *Porphyromonadaceae*, *Lachnospiraceae*, *Shewanellaceae*, *Peptococcaceae*, and *Deferribacteraceae*. The feces of control Jax mice had more *Bifidobacteriaceae* and S24_7. These data indicate significant differences were observed not only in the loss or gain of specific bacterial taxa, but also in the relative abundance of members of the GI microbial community structures between Jax and Tac B6 females.

### The gastrointestinal microbial structures between the vendors and relative abundances of bacteria were differentially perturbed by *H*. *pylori* infection

**Figure 6:**
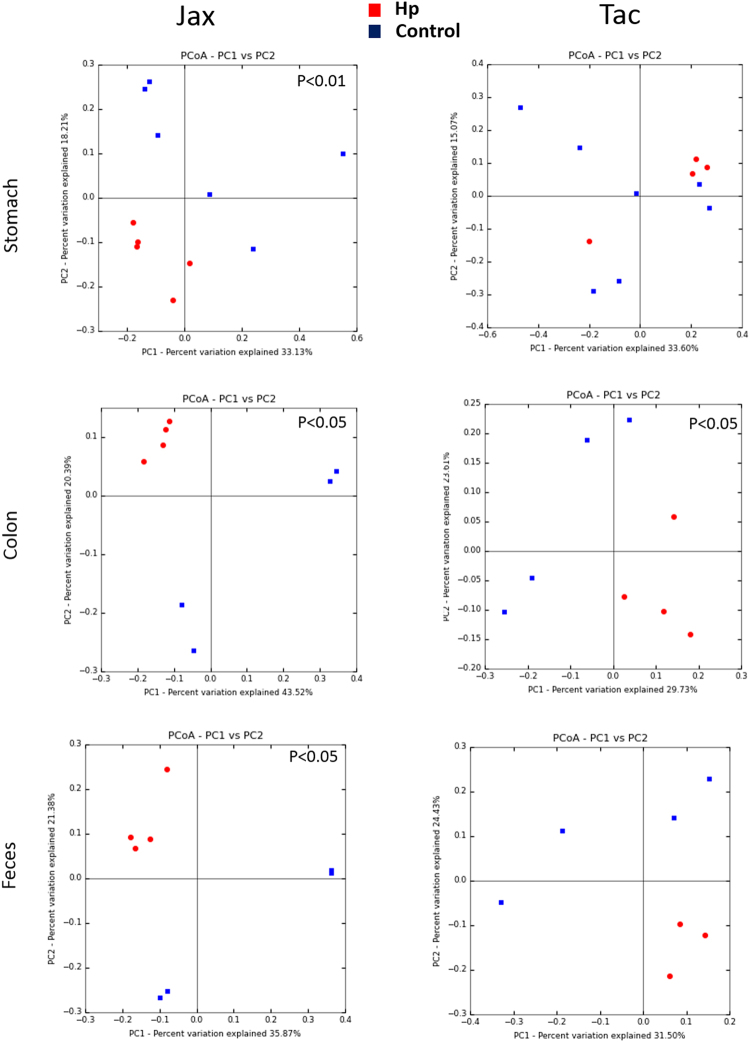
The effects of *H*. *pylori* infection on gastric, colonic and fecal microbiomes of B6 females. Principal coordinates analysis (PCoA) (the unweighted UniFrac) was performed based on bacterial 16S rRNA gene sequences at an even sample depth (2743). Samples from PMSS1-infected (blue) and sham control (red) mice are represented as colored circles. P values are calculated on the corresponding unweighted UniFrac distance matrix using ADONIS test and <0.05 is considered significant.

A previous report indicated that *H*. *pylori* infection significantly altered the microbial communities in the stomach, the colon and the feces of female Jax B6 mice^14^, whereas another study reported that only select groups of bacteria but not overall gastric microbial community structures of female Tac B6 mice were significantly perturbed by *H*. *pylori* infection^30^. Evaluating the effect of *H*. *pylori* infection on the alpha diversity of mice demonstrated that *H*. *pylori* infection did not alter the diversity in the stomach, colon or feces of either Tac or Jax mice. While *H*. *pylori* infection did not significantly alter the diversity in B6 mice, PCoA analyses (unweighted UniFrac using the ADONIS test) indicated that *H*. *pylori* infection significantly altered gastric, colonic and fecal microbial community structures of Jax B6 mice (P < 0.01, 0.05, 0.05, respectively). However, in Tac B6 mice only the microbial community structure of the colon was significantly perturbed (P < 0.05), while the communities of the stomach and feces did not exhibit a change in beta diversity (Fig. 6).

Compared to sham controls, *H*. *pylori*-infected Jax B6 mice had increased abundance of *Helicobacteraceae* (*H*. *pylori*) in the stomach and *Dehalobacteriaceae* in the colon and feces. *H*. *pylori-*infected Jax mice had decreased abundance of *Bifidobacteriaceae* (stomach, colon and feces), *Anaeroplasmataceae* (stomach and feces), unassigned bacterial groups and *Clostridiaceae* (stomach), *Coribacteriaceae* (colon) and *Turicibacteriaceae* (feces) (Table S2).

Despite the fact that overall microbial community structures in the stomach and feces of Tac B6 mice were not significantly altered by *H*. *pylori* infection, there were significant differences in the relative abundances of several families in the stomach, colon and feces. *H*. *pylori*-infected mice had increased abundance of *Helicobacteraceae* (stomach), *Lactobacillaceae* (colon and feces) and *Mogibacteriaceae* (feces) compared to uninfected Tac mice. Uninfected Tac mice had higher levels of *Turicibacteraceae* (stomach and feces), *Peptostreptococcaceae* (stomach) and *Clostriadiaceae* (stomach) relative to *H*. *pylori*-infected Tac mice (Table S2). These data indicate that the bacterial communities along the GI have differential responses dependent on the original microbiota present prior to *H*. *pylori* infection.

### Differences in the gastric microbial community structures between Jax and Tac B6 females potentially influence bacterial function based on ‘predictive metagenomics’

Enrichment of select bacterial genes has been associated with health and disease in the host^29^. Using PICRUSt, we were able to predict functional differences between Tac and Jax mice *in silico*. 92 KEGG pathways in the stomach were differentially represented between Jax (49 pathways increased) and Tac B6 (43 pathways increased) mice (Tables 1, S3). These affected KEGG pathways predominantly belong to four KEGG modules: Metabolism, Genetic information processing, Organismal systems and Environmental information processing. Interestingly, the increased KEGG pathways in the stomach of Jax B6 mice involve amino acid metabolism, biosynthesis of secondary metabolites, endocrine system, energy metabolism and glycan biosynthesis and metabolism, whereas the increased KEGG pathways in the stomach of Tac B6 females play a role in lipid metabolism, xenobiotics biodegradation and metabolism, and metabolism of cofactors and vitamins. In addition, antigen processing, NOD-like receptor signaling pathway, epithelial cell signaling in *H*. *pylori* infection were significantly promoted in the stomach of Jax mice compared to Tac mice, which was in line with higher colonization levels of *H*. *pylori* in Jax mice compared Tac mice. *In silico* prediction indicates that the gastric microbial community structures between Jax and Tac mice promote different bacterial functions, which could potentially modulate *H*. *pylori*-induced pathophysiology of mice from different vendors. Despite the difference in the colonic microbial community structures between Jax and Tac B6 mice, significant differences in all KEGG pathways of the colon were not detected between Jax and Tac B6 mice.

**Table 1:** Promotion of distinct KEGG pathways *in silico* between Jax (in bold) and Tac B6 mice based on their gastric microbial community structures.

Level_1	Level_2	Level_3	Jax: mean rel. freq. (%)	Tac: mean rel. freq. (%)	p-values	p-values (corrected)
Metabolism	Biosynthesis of Other Secondary Metabolites	**Isoquinoline alkaloid biosynthesis**	0.03404	0.02201	0.00112	0.01017
Metabolism	Biosynthesis of Other Secondary Metabolites	**Novobiocin biosynthesis**	0.09762	0.06572	0.00417	0.02173
Metabolism	Biosynthesis of Other Secondary Metabolites	**Phenylpropanoid biosynthesis**	0.15779	0.102	0.00435	0.02231
Metabolism	Biosynthesis of Other Secondary Metabolites	**Tropane, piperidine and pyridine alkaloid biosynthesis**	0.077	0.05614	0.00847	0.03386
Organismal Systems	Endocrine System	**Adipocytokine signaling pathway**	0.03852	0.02368	0.00249	0.01512
Organismal Systems	Endocrine System	**Insulin signaling pathway**	0.054	0.03361	0.01225	0.04564
Organismal Systems	Endocrine System	**Progesterone-mediated oocyte maturation**	0.03291	0.02043	0.00196	0.01338
Metabolism	Energy Metabolism	**Carbon fixation in photosynthetic organisms**	0.56654	0.52247	0.00563	0.02676
Metabolism	Energy Metabolism	**Energy metabolism_Unclassified**	0.83812	0.66251	0.00076	0.00829
Metabolism	Energy Metabolism	**Methane metabolism**	1.20874	1.11594	0.00767	0.03186
Metabolism	Energy Metabolism	**Oxidative phosphorylation**	1.06587	0.96249	0.00219	0.01438
Metabolism	Glycan Biosynthesis and Metabolism	**Glycosaminoglycan degradation**	0.06854	0.01885	0.00124	0.01066
Metabolism	Glycan Biosynthesis and Metabolism	**Glycosphingolipid biosynthesis - ganglio series#**	0.04443	0.00841	0.00152	0.01217
Metabolism	Glycan Biosynthesis and Metabolism	**Glycosphingolipid biosynthesis - globo series**	0.12934	0.06733	0.00350	0.01913
Metabolism	Glycan Biosynthesis and Metabolism	**N-Glycan biosynthesis**	0.01782	0.00909	0.00734	0.03166
Metabolism	Glycan Biosynthesis and Metabolism	**Other glycan degradation**	0.32593	0.17931	0.01301	0.04688
Metabolism	Lipid Metabolism	Biosynthesis of unsaturated fatty acids	0.13432	0.17545	0.00221	0.01420
Metabolism	Lipid Metabolism	Fatty acid biosynthesis	0.37939	0.50576	0.00002	0.00105
Metabolism	Lipid Metabolism	Fatty acid metabolism	0.30793	0.3885	0.00987	0.03901
Metabolism	Lipid Metabolism	Lipid biosynthesis proteins	0.54039	0.59328	0.00043	0.00562
Metabolism	Lipid Metabolism	Primary bile acid biosynthesis	0.04784	0.07433	0.00583	0.02621
Metabolism	Lipid Metabolism	Secondary bile acid biosynthesis	0.04771	0.07408	0.00581	0.02684
Metabolism	Xenobiotics Biodegradation and Metabolism	Aminobenzoate degradation	0.16468	0.19258	0.00213	0.01423
Metabolism	Xenobiotics Biodegradation and Metabolism	Benzoate degradation	0.2908	0.36408	0.00125	0.01049
Metabolism	Xenobiotics Biodegradation and Metabolism	Bisphenol degradation	0.08076	0.088	0.00316	0.01816
Metabolism	Xenobiotics Biodegradation and Metabolism	Caprolactam degradation	0.07281	0.11418	0.01229	0.04530
Metabolism	Xenobiotics Biodegradation and Metabolism	Chloroalkane and chloroalkene degradation	0.20301	0.25655	0.00013	0.00299
Metabolism	Xenobiotics Biodegradation and Metabolism	Dioxin degradation	0.0772	0.10618	0.01061	0.04143
Metabolism	Xenobiotics Biodegradation and Metabolism	Drug metabolism - cytochrome P450	0.06089	0.10521	0.00078	0.00824
Metabolism	Xenobiotics Biodegradation and Metabolism	Metabolism of xenobiotics by cytochrome P450	0.05715	0.10393	0.00047	0.00547
Metabolism	Xenobiotics Biodegradation and Metabolism	Naphthalene degradation	0.16058	0.21657	0.00018	0.00360
Metabolism	Xenobiotics Biodegradation and Metabolism	Polycyclic aromatic hydrocarbon degradation	0.09134	0.12857	0.00028	0.00509

## Discussion

C57BL/6 mice (B6) have been widely used in biomedical research due to the in-depth knowledge of their genetic profile and their use in an array of transgenic mouse strains. In this study, we have demonstrated that there are different responses to *H*. *pylori* infection in female B6 mice obtained from the Jackson Laboratory (Jax) versus Taconic Biosciences (Tac). The infected Jax B6 mice had higher colonization levels of gastric *H*. *pylori*, elevated transcription of gastric *Il-1β* and *Il-17A* compared to the infected Tac counterparts. In addition, there were more severe mucosal metaplasia of the stomach and stronger Th1-associated IgG2c response to *H*. *pylori* in the infected Tac B6 mice compared to the infected Jax B6 mice. Further, microbiome data revealed that the abundance and evenness of gastric, colonic and fecal microbial compositions significantly differed between Jax and Tac B6 strains. *H*. *pylori* infection significantly altered the microbial community structures at all the tested sites (stomach, colon and feces) in Jax B6 females, but only altered the colonic microbial community structures in the colon of Tac counterparts. These findings demonstrated that a particular mouse strain with different microbiota can develop different host immune responses and pathological alterations induced by *H*. *pylori*.

Mucous metaplasia, which is defined as the expression of intestinal-type mucin within the gastric corpus, can spontaneously develop in some mouse strains and is aggravated by *H*. *pylori* infection^22,31^. In this study, we found that *H*. *pylori* infection induced more severe gastric mucous metaplasia in Tac B6 mice compared to Jax B6 mice. This finding was further supported by the increased proinflammatory Th1-type IgG2c response to *H*. *pylori* and lower colonization efficiency of *H*. *pylori* in Tac B6 over Jax B6, which is consistent with our previous study showing that there is an inverse relationship between *H*. *pylori* colonization levels and gastric disease severity. These data suggest that more severe mucous metaplasia developed in *H*. *pylori*-infected Tac B6 compared to *H*. *pylori*-infected Jax B6 mice is attributable directly or indirectly to *H*. *pylori* infection^12^. Interestingly, severity of this lesion was also qualitatively correlated with enhancement of expression of Tff2 and GSII, makers for both mucous metaplasia and precancerous spasmolytic polypeptide-expressing metaplasia (SPEM), in the gastric corpus of *H*. *pylori*-infected B6^22,24^. A recent study, co-expression of GSII and clustein were elevated in the Kras-activated SPEM glands of the stomach of Mist1-Kras mice^24^. Increased expression of GSII and clustein was also found in the gastric corpus of gerbils infected with *H*. *pylori* strain 7.13; GSII and clusterin were expressed in SPEM developed in the base of oxyntic glands and in the tips of branched and dilated lesions as well as invasive glands respectively^32^. Our results and these recent findings suggest that some of Tff2/GSII-positive glands in the regions undergoing mucous metaplasia are consistent with SPEM in the infected B6 mice^22^. The role of SPEM as a premalignant marker could be further studied by using the *H*. *pylori*-infected Tac B6 mice and Jax B6 mice for a longer duration, since it has already known that high-grade hyperplasia and dysplasia develop in Tac B6 infected with *H*. *pylori* SS1 for 11 months^12^.

It has been established that Infγ-expressing Th1 cells induce IgG class switching in B cells to IgG2a production^33,34^. Our previous studies showed that the development of serum Th1-associated IgG2c (equivalent to IgG2a) response to *H*. *pylori* infection were positively associated with *H*. *pylori* gastric pathology in B6 and Tff2^−/−^ mice (B6 X Sv129)^12,22,35^. Additionally, co-infection with enterohepatic *H*. *bilis* in *H*. *pylori* SS1-infected female B6 mice attenuated gastric pathology (GHAI) compared with mono-*H*. *pylori* infected B6 mice in concert with lower levels of both serum Th1-associated IgG2c and gastric *Infγ* mRNA^12^. In this study, we showed that there was a higher IgG2c response to PMSS1 infection in Tac B6 mice vs Jax mice, whereas mRNA levels of gastric *Ifn-γ* in PMSS1-infected Tac mice were relatively higher with no statistical significance than those in PMSS1-infected Jax mice at 4 months post infection (MPI). The relatively higher level of gastric *Ifn-γ* mRNA in PMSS1-infected Tac mice vs Jax mice suggests that the elevated Th1 cell response to *H*. *pylori* infection in Tac B6 mice could occur early in *H*. *pylori* infection, and promoted a Th1-associated IgG2c response; this elevation of the *H*. *pylori*-induced Th1 response in the stomach may be decreased due to gradual clearance of gastric *H*. *pylori* organisms during the course of *H*. *pylori* infection. This hypothesis is supported by current and previous studies. First, 4 out of 10 *H*. *pylori*-infected Tac B6 mice were *H*. *pylori*-free and there was a relatively lower colonization level of gastric *H*. *pylori* in *H*. *pylori*-positive Tac mice compared to that in *H*. *pylori*-positive Jax mice. Second, our previous findings indicate that the gastric Th1 response but not the Th17 response plays an important role in decreasing *H*. *pylori* colonization in female B6 mice, since concurrent infection of *H*. *hepaticus* in *H*. *pylori*-infected B6 promoted the Th17 response and aggravated *H*. *pylori* gastric pathology, but was associated with a higher *H*. *pylori* colonization at 6 MPI when compared with the mice infected with *H*. *pylori* alone^13^. Third, the gradual clearance of *H*. *pylori* colonization in Tac B6 mice noted in our study is suggested by the results that levels of *Ifn-γ* expression and the IgG responses (total IgG, Th1-associatd Th1 IgG2c and Th2-associated IgG1) as well as the GHAI scores between *H*. *pylori*-negative and –positive mice from the *H*. *pylori*-infected Tac group were comparable.

It was previously reported that B6 mice from different vendors developed different pathological and immunological response to *H*. *pylori* infection^30,36^. PMSS1-infected CLR B6 mice developed more severe antral pathology compared with PMSS1-infeced Jax B6 counterparts^36^. In another study, *H*. *pylori* SS1-infected B6 mice from Tac developed more severe gastric inflammation compared to *H*. *pylori* SS1-infected B6 mice from the Charles River Laboratories (CRL)^30^. Tac and CRL mice appeared to contain different gastric microbiota as evidenced by different levels of two *Lactobacillus spp*., ASF360 and ASF361, in the stomachs^30^. Additionally, antibiotic-pretreated Tac B6 mice, with the gastric microbiota compositionally distinct from the non-antibiotic treated mice, developed weaker CD4+ cell responses to *H*. *pylori*, indicating that the residing GI microbiota are influenced by *H*. *pylori*-induced gastric inflammation^30^. We found that the gastric bacterial community structures were significantly different between Jax and Tac B6 mice. These differences appeared not to influence the initial colonization levels of *H*. *pylori* PMSS1 in B6 mice between Jax and Tac, since there was no statistical significant difference in the serum levels of total IgG and IgG1 (a surrogate biomarker for *H*. *pylori* infection in mice and humans) as well as the average levels of *H*. *pylori* colonization between *H*. *pylori* PMSS1-infected Jax and Tac B6 mice at 16 WPI. Lower colonization levels of *Lactobacillaceae* and absence of *Akkemansia* in the stomach of Jax B6 mice, both of which have probiotic potential, may have contributed to the higher colonization levels of *H*. *pylori* during the course of *H*. *pylori* infection and significant perturbation of the gastric microbial structure. This in turn could have promoted higher transcript levels of gastric *Il-17A*, *Il-1β* and *RegIIIγ* compared to PMSS1-infected Tac mice. Additionally, *in silico* predictive metagenomic analysis indicated that the differences in the gastric bacterial community structures could potentially modulate functions of distinct KEGG pathways between Jax and Tac mice. The pathways involved in metabolism of energy, amino acids and biosynthesis of secondary metabolites were promoted in the stomach of Jax mice, whereas the KEGG pathways involved in lipid metabolism, xenobiotics biodegradation and metabolism of cofactors and vitamins were upregulated in the stomach of Tac B6 mice. The functional disparities of these KEGG pathways could influence physiology of the GI tract, resulting in differential responses to *H*. *pylori* infection. Thus, it is plausible that the compositional and abundance differences in the gastric microbiomes play a major role in the differential host responses to *H*. *pylori* infection between Jax and Tac B6 mice.

It has been documented that effects of *H*. *pylori* infection on the GI bacterial community structures are variable in different mouse strains. In INS-GAS mice, *H*. *pylori* SS1 infection increased the population of *Bacteriodetes* with reduction of *Firmicutes* in the stomach, but had minimal effects on colonic and cecal microbial structures compared to sham controls^10^. Rolig *et al*. reported that *H*. *pylori* SS1 infection did not significantly alter the overall gastric bacterial community structures in Tac mice except for select bacterial groups; *Bacilli*, *Bacteroidetes* and *Proteobacteria* were decreased with increases of *Clostridia*, *Helicobacter* and *Verrucomicrobia* noted in *H*. *pylori*-infected mice^30^. A recent study reported that *H*. *pylori* PMSS1 infection in Jax mice significantly affected not only the gastric, but also ileal, cecal and fecal bacterial structures and relative bacterial abundances^14^. Consistent with these findings, PMSS1 infection in Jax B6 mice in our study significantly affected the β diversity of the gastric, colonic and fecal bacterial community structures; *H*. *pylori* infection, however, did not alter the β diversity of gastric and fecal bacterial compositions in Tac mice. Additionally, the family S24-7 of *Bacteroidales* is a predominant flora in both the Jax B6 mice in this study and the Jax B6 mice used by Kienesberger *et al*.^14^. It is worth noting that SFB (*Canaditus* Arthromitus) and *Akkemansia*, which were absent from Jax mice in our study, were also noted to be present in the Jax mice of Kienesberger *et al*.^14^. Since SFB are detected only in Tac B6, but not in Jax B6 in our studies and other groups^16,26,37^, SFB and possibly *Akkemansia* were likely introduced into these Jax B6 cohorts while housed at their facility^14^. These findings collectively indicate that the ability of *H*. *pylori* infection to perturb the GI bacterial community structures varied in B6 mice from different vendors, which is likely due to host susceptibility, the GI microbial community structures of the host, husbandry and housing conditions.

SFB in Tac B6 mice of our study persistently and predominantly colonized the ileum irrespective of infection status, which was associated with over 100-fold increase of ileal *Il-17A* mRNA, but not *Tnfα* and *Il-1β* mRNA when compared to both *H*. *pylori* infected and control Jax mice. The mRNA levels of ileal *Ifnγ* in all Tac B6 mice was also increased, which may be part of the host’s response to maintain the homoeostasis of a Th17 cell population^38,39^. However, more severe gastric mucous metaplasia and reduction of *H*. *pylori* colonization levels in Tac B6 mice did not appear to directly result from the presence of SFB, because the level of gastric *Il-17A* mRNA was comparable between Tac B6 mice and Jax B6 controls. However, *H*. *pylori* infection increased SFB colonization levels in the large bowel, which is consistent with our previous finding in Tac Swiss Webster mice infected with *H*. *hepaticus*^26^. Given that Th17 cells can act as a host defense against infection of a wide spectrum of pathogenic microorganisms^40^, SFB may play an important role in shaping and maintaining the gastrointestinal microbial structures in Tac B6 mice, which were compositionally distinct from those in Jax B6 mice, thereby indirectly affecting the pathological and immunological responses of Tac B6 mice to *H*. *pylori* infection.

Taken together, we have demonstrated that B6 mice from Jax and Tac contain distinct GI microbial community structures and develop differential immune, pathological and microbial responses to *H*. *pylori* infection. Differences in the GI microbiomes between Jax and Tac B6 mice may result from different housing husbandry practices between the vendors as well as phenotypic and genetic variations between these C57BL/6 sub-strains^29,41^. The interplay between *H*. *pylori* and the GI microbiota and the resulting pathological outcome are becoming increasingly evident in both mice and humans. Our findings further emphasize the importance of defining the source of the animal models being used in a particular study given that the same inbred strain of mouse can have the different GI microbial communities which can dictate different responses to a given experimental treatment.

## Materials and Methods

### Mice and infection

Mice were maintained in a facility accredited by the Association for the Assessment and Accreditation of Laboratory Animal Care. All animal care and experimental procedures were approved by the Massachusetts Institute of Technology Committee on Animal Care and Use in accordance with the Guide for the Care and Use of Laboratory Animals. Fifteen 4-week-old female C57BL/6 (B6) mice were purchased from Taconic Biosciences (Tac) (Germantown, NY) and the Jackson Laboratory (Jax) (Bar Harbor, ME), respectively. The mice were free of known murine viruses, *Helicobacter* spp. and parasites, and were maintained in static microisolator cages and fed a diet (ProlabRMH3000) from PMI Nutrition International (Richmond, IN**)**. After being in quarantine for a week, 10 mice from each vendor were inoculated with *H*. *pylori* PMSS1 (containing a functional CagA), whereas 5 control mice of each vendor, which were housed in a separate cubicle, were sham-dosed with vehicle. Mice received 0.2 ml of fresh inocula (~2 × 10^8^ organisms) by gastric gavage every other day for a total of three inoculations.

### Necropsy and histopathology

Fecal samples from each B6 mouse were collected prior to inoculation, 2, 4, 8 and 16 weeks post inoculation with *H*. *pylori* (WPI). All infected and control mice from each group were euthanized at 16 WPI. Immediately after euthanasia of the mice with CO_2_, stomach samples from the lesser curvature extending from the squamous forestomach through the proximal duodenum were collected and processed as previously described^31,42^. Gastric lesions were graded by a comparative pathologist (S. Muthupalani) blinded to sample identity on an ascending scale from 0 to 4 for inflammation, epithelial defects, atrophy, hyperplasia, pseudopyloric metaplasia, dysplasia, and mucous metaplasia as defined elsewhere^31^. A gastric histologic activity index (GHAI) was generated by combining scores for all criteria. Additionally, the cecocolic junction and colon were embedded in paraffin, sectioned at 5 µm and stained with hematoxylin and eosin for histologic evaluation using the same scoring system as previously described^43^.

### Preparation of DNA and RNA from tissue and fecal samples

Contents in the stomach and intestine were removed by rinsing with sterile PBS. A piece of gastric tissue containing antrum and corpus and one-cm segments of ileum, cecum, and colon for isolation of RNA or DNA were collected and frozen in liquid nitrogen immediately after sampling and stored at −70 °C prior to use. Total DNA from the collected tissues and fecal samples was isolated using the High Pure PCR Template Preparation Kit (Roche, Applied Science, Indianapolis, IN) and using the QIAamp Fast DNA Stool Mini Kit according to the supplier’s protocol (Qiagen Inc., Valencia, CA), respectively. Total RNA from gastric and intestinal tissues was isolated using Trizol Reagents following the supplier’s procedure (Invitrogen, Carlsbad, CA). Purity of the prepared DNA or RNA were analyzed using Nanodrop 2000C (Thermo Scientific, Wilmington, DE).

### Immunohistochemistry

Stomach sections from all the groups were stained with specific polyclonal rabbit anti- Tff2 IgG^22^. Additionally, gastric mucous neck cells were identified using fluorescein isothiocyanate (FITC)-conjugated Griffonia (Bandeiraea) simplicifolia lectin II (GSII) and slides were mounted with Vectashield plus DAPI (4,6-diamidino-2-phenylindole) (Vector Laboratories, Burlingame, CA). Routine immunohistochemistry was performed as described previously with minor modifications^22^. The antigens were retrieved at a low and high pH, depending on the antibodies, by incubation for 45 minutes at 95 °C, and the slides were stained using an automated stainer (Lab Vision Autostainer 360; Thermo Fisher Scientific, Waltham, MA, USA) sequentially using a 10-min Ultravision peroxidase block, a 30-min Rodent block R, primary antibodies for 30 minutes, followed by appropriate secondary polymer based anti-rabbit IgG, and developed with diaminobenzidine and counterstained with H&E. Slides were dehydrated and cleared, and then coverslipped for qualitative assessment of the expression patterns of the various markers.

### ELISA

Methods used were as previously published^12^.

### 16S rRNA gene sequencing and analysis

DNA extracted from gastric tissues, colon and fecal samples was amplified using universal primers of F515 (GTGCCAGCMGCCGCGGTAA) and R806 (GGACTACHVGGGTWTCTAAT) to target the V4 regions of bacterial 16S rRNA^44^. Individual samples were barcoded and pooled to construct the sequencing library, followed by sequencing with an Illumina MiSeq instrument to generate pair-ended 250 × 250 reads. Overlapping pair-end reads were aligned using PEAR^45^. Paired reads shorter than 270 bp were filtered out. Subsequent analysis and normalization were performed using QIIME 1.9.1^46^ within the MicrobiomeHelper v. 2.0.0 virtual box^47^.Chimeric sequences were removed by comparison to the database Bacteria_RDP_trainset15_092015.fa. Operational taxonomic units (OTUs) present at less than 34 reads were removed. The average number of reads for the gastric, colonic and fecal samples were 31,873, 272,885 and 139,673 reads, respectively. For all samples, the median number of reads/sample was 131,970 with a minimum of 2,743 reads/sample in a gastric sample and a maximum of 366,949 reads/sample in a colonic sample. Microbial communities were compared by using UniFrac. Sequences were grouped into OTUs at 97% sequence similarity using uclust. Taxonomy was assigned using Ribosomal Database Project (RDP) classifier against GreenGenes database, and sequences were aligned and phylogenetic tree was built from reference sequences using FastTree. An OTU table showing counts of each OTU in each sample was produced. To control for differences in sequencing depth, OTU tables were rarified at a single sequencing depth^48,49^. Alpha diversity was determined using Observed Species and Shannon index and was represented as rarefaction curves. Significant differences in alpha diversity were calculated using QIIME using a non-parametric two-sample t-test with p-values < 0.05 considered significant. Beta diversity was determined using unweighted UniFrac^50^ and the results presented as principal coordinate axis (PCoA) plots. R (version 3.4.1 at http://www.R-project.org/) and libraries ape (4.1), ggplot2 (2.2.1), vegan (2.4.3 at https//CRAN.R-project.org/package=vegan), and phyloseq (1.19.1) were used to perform statistical analyses of the unweighted UniFrac distance matrices using ADONIS^51–53^, which performs analysis of variance using distance matrices. P-values < 0.05 were considered significant. The OTU’s were summarized at different taxonomic levels from phylum to genus and differences in the relative abundance at different taxonomic levels were determined using ANOVA with a false discovery rate of 5%. Significant differences in relative abundance of select bacterial taxa was analyzed using Linear discriminant analysis effect size (LEfSe)^54^. Using LEfSe, we compared the effects of vendors among uninfected controls or the effect of infection in each tissue site for each vendor separately. Statistical significance was determined using the Kruskal-Wallis test with a post-hoc pairwise Wilcoxon test to compare subclasses. Samples with P-values < 0.05 for both tests were then screened for effect size using a threshold of 2 for the logarithmic linear discriminant analysis score computed by LEfSE. *In silico* prediction for capability of KEGG pathways was performed using PICRUSt 1.1^55^. STAMP software was used to analyze the significance of level 3 KEGG pathways in the stomach using a two-sided Welch’s t-test and the Benjamini-Hochberg procedure for multiple test correction^56^. Pathways with corrected P-values < 0.05 were considered significant.

### Quantification of *H. pylori* and SFB

Colonization levels of gastric *H*. *pylori* PMSS1 and SFB in stomach, ileum, cecum, colon and feces were quantified using qPCR in the 7500 FAST Real-time PCR System (Life Technologies) as previously described^26^. Copy numbers of target bacteria were then normalized to μg of mouse chromosomal DNA whose quantities in the samples were measured by qPCR using the 18S rRNA gene-based primers and probe mixture (Life Technologies).

### Expression of select cytokines

For relative mRNA quantitation of selected genes, total RNA from stomach and ileum of B6 mice was prepared using Trizol reagent according to manufacturer’s recommendations (Invitrogen, Carlsbad, CA). Five μg of total RNA from each sample was converted into cDNA using the High Capacity cDNA Archive kit (Life Technologies). Levels of *Ifnγ*, *Tnfα*, *Il-1β*, *Il-17A*, *Il-22*, *RegIIIγ*, *Il-10* and *Foxp3* mRNA in the gastric and ileal tissues were measured by qPCR using commercial primers and probes (TaqMan Gene Expression Assays) in the 7500 FAST Real-time PCR System. Transcript levels were normalized to the endogenous control glyceraldehyde-3-phosphate dehydrogenase mRNA (*Gapdh*) and expressed as fold change in reference to sham controls (defined as 0) using the comparative threshold cycle (*CT*) method (Applied Biosystems User Bulletin No. 2).

### Statistical analysis

Data on the levels of SFB, *H*. *pylori* and cytokine expression were analyzed among multiple groups using a Kruskal-Wallis test and between two groups using an unpairedstudent *t* test. The Mann-Whitney *U* test was used for analyzing the GHAI scores between the groups. Values of P < 0.05 were considered significant.

## Electronic supplementary material

Supplemental information

## References

[1] Fox JG, Wang TC (2007). Inflammation, atrophy, and gastric cancer. J Clin Invest.

[2] Graham DY (2015). Helicobacter pylori update: gastric cancer, reliable therapy, and possible benefits. Gastroenterology.

[3] McColl KE (2010). Clinical practice. Helicobacter pylori infection. N Engl J Med.

[4] Yamaoka Y, Graham DY (2014). Helicobacter pylori virulence and cancer pathogenesis. Future Oncol.

[5] Fox JG, Wang TC (2014). Dietary factors modulate Helicobacter-associated gastric cancer in rodent models. Toxicologic pathology.

[6] Amieva M, Peek RM (2016). Pathobiology of Helicobacter pylori-Induced Gastric Cancer. Gastroenterology.

[7] Sheh A, Fox JG (2013). The role of the gastrointestinal microbiome in Helicobacter pylori pathogenesis. Gut microbes.

[8] Cao L, Yu J (2015). Effect of Helicobacter pylori Infection on the Composition of Gastric Microbiota in the Development of Gastric Cancer. Gastrointestinal tumors.

[9] Yang I (2016). Different gastric microbiota compositions in two human populations with high and low gastric cancer risk in Colombia. Scientific reports.

[10] Lofgren JL (2011). Lack of commensal flora in Helicobacter pylori-infected INS-GAS mice reduces gastritis and delays intraepithelial neoplasia. Gastroenterology.

[11] Lertpiriyapong K (2014). Gastric colonisation with a restricted commensal microbiota replicates the promotion of neoplastic lesions by diverse intestinal microbiota in the Helicobacter pylori INS-GAS mouse model of gastric carcinogenesis. Gut.

[12] Lemke LB (2009). Concurrent Helicobacter bilis infection in C57BL/6 mice attenuates proinflammatory Helicobacter pylori-induced gastric pathology. Infection and immunity.

[13] Ge Z (2011). Coinfection with Enterohepatic Helicobacter species can ameliorate or promote Helicobacter pylori-induced gastric pathology in C57BL/6 mice. Infection and immunity.

[14] Kienesberger S (2016). Gastric Helicobacter pylori Infection Affects Local and Distant Microbial Populations and Host Responses. Cell reports.

[15] O’Rourke JL, Lee A (2003). Animal models of Helicobacter pylori infection and disease. Microbes and infection / Institut Pasteur.

[16] Ivanov II (2009). Induction of intestinal Th17 cells by segmented filamentous bacteria. Cell.

[17] Suzuki K (2004). Aberrant expansion of segmented filamentous bacteria in IgA-deficient gut. Proceedings of the National Academy of Sciences of the United States of America.

[18] Ohashi Y, Hiraguchi M, Ushida K (2006). The composition of intestinal bacteria affects the level of luminal IgA. Bioscience, biotechnology, and biochemistry.

[19] Gaboriau-Routhiau V (2009). The key role of segmented filamentous bacteria in the coordinated maturation of gut helper T cell responses. Immunity.

[20] Goto Y (2014). Segmented filamentous bacteria antigens presented by intestinal dendritic cells drive mucosal Th17 cell differentiation. Immunity.

[21] Lecuyer E (2014). Segmented filamentous bacterium uses secondary and tertiary lymphoid tissues to induce gut IgA and specific T helper 17 cell responses. Immunity.

[22] Fox JG (2007). Accelerated progression of gastritis to dysplasia in the pyloric antrum of TFF2-/- C57BL6 x Sv129 Helicobacter pylori-infected mice. Am J Pathol.

[23] Peterson AJ (2010). Helicobacter pylori infection promotes methylation and silencing of trefoil factor 2, leading to gastric tumor development in mice and humans. Gastroenterology.

[24] Choi E, Hendley AM, Bailey JM, Leach SD, Goldenring JR (2016). Expression of Activated Ras in Gastric Chief Cells of Mice Leads to the Full Spectrum of Metaplastic Lineage Transitions. Gastroenterology.

[25] Zhuang Y (2015). A pro-inflammatory role for Th22 cells in Helicobacter pylori-associated gastritis. Gut.

[26] Ge Z, Feng Y, Woods SE, Fox JG (2015). Spatial and temporal colonization dynamics of segmented filamentous bacteria is influenced by gender, age and experimental infection with Helicobacter hepaticus in Swiss Webster mice. Microbes and infection / Institut Pasteur.

[27] Cho I, Blaser MJ (2012). The human microbiome: at the interface of health and disease. Nature reviews. Genetics.

[28] Abreu MT, Peek RM (2014). Gastrointestinal malignancy and the microbiome. Gastroenterology.

[29] Lynch SV, Pedersen O (2016). The Human Intestinal Microbiome in Health and Disease. N Engl J Med.

[30] Rolig AS, Cech C, Ahler E, Carter JE, Ottemann KM (2013). The degree of Helicobacter pylori-triggered inflammation is manipulated by preinfection host microbiota. Infection and immunity.

[31] Rogers AB (2005). Helicobacter pylori but not high salt induces gastric intraepithelial neoplasia in B6129 mice. Cancer research.

[32] Shimizu T (2016). Characterization of progressive metaplasia in the gastric corpus mucosa of Mongolian gerbils infected with Helicobacter pylori. The Journal of pathology.

[33] Snapper CM, Paul WE (1987). Interferon-gamma and B cell stimulatory factor-1 reciprocally regulate Ig isotype production. Science.

[34] Toellner KM (1998). T helper 1 (Th1) and Th2 characteristics start to develop during T cell priming and are associated with an immediate ability to induce immunoglobulin class switching. J Exp Med.

[35] Sheh A (2010). Mutagenic potency of Helicobacter pylori in the gastric mucosa of mice is determined by sex and duration of infection. Proceedings of the National Academy of Sciences of the United States of America.

[36] Sigal M (2015). Helicobacter pylori Activates and Expands Lgr5(+) Stem Cells Through Direct Colonization of the Gastric Glands. Gastroenterology.

[37] Arias CA, Murray BE (2012). The rise of the Enterococcus: beyond vancomycin resistance. Nature reviews.

[38] Jiang H, Chess L (2004). An integrated view of suppressor T cell subsets in immunoregulation. J Clin Invest.

[39] Harrington LE (2005). Interleukin 17-producing CD4+ effector T cells develop via a lineage distinct from the T helper type 1 and 2 lineages. Nature immunology.

[40] Curtis MM, Way SS (2009). Interleukin-17 in host defence against bacterial, mycobacterial and fungal pathogens. Immunology.

[41] Simon MM (2013). A comparative phenotypic and genomic analysis of C57BL/6J and C57BL/6N mouse strains. Genome biology.

[42] Erdman SE (2003). CD4(+)CD25(+) regulatory lymphocytes require interleukin 10 to interrupt colon carcinogenesis in mice. Cancer research.

[43] Erdman SE (2005). CD4+CD25+ regulatory lymphocytes induce regression of intestinal tumors in ApcMin/ + mice. Cancer research.

[44] Caporaso JG (2011). Global patterns of 16S rRNA diversity at a depth of millions of sequences per sample. Proceedings of the National Academy of Sciences of the United States of America.

[45] Zhang J, Kobert K, Flouri T, Stamatakis A (2014). PEAR: a fast and accurate Illumina Paired-End reAd mergeR. Bioinformatics.

[46] Caporaso JG (2010). QIIME allows analysis of high-throughput community sequencing data. Nature methods.

[47] Comeau, A. M., Douglas, G. M. & Langille, M. G. Microbiome Helper: a Custom and Streamlined Workflow for Microbiome Research. *mSystems* **2**, 10.1128/mSystems.00127-16 (2017). 10.1128/mSystems.00127-16 PMC5209531 28066818

[48] Hamady M, Knight R (2009). Microbial community profiling for human microbiome projects: Tools, techniques, and challenges. Genome research.

[49] Kuczynski J (2010). Direct sequencing of the human microbiome readily reveals community differences. Genome biology.

[50] Lozupone C, Hamady M, Knight R (2006). UniFrac–an online tool for comparing microbial community diversity in a phylogenetic context. BMC bioinformatics.

[51] Paradis E, Claude J, Strimmer K (2004). APE: Analyses of Phylogenetics and Evolution in R language. Bioinformatics.

[52] McMurdie PJ, Holmes S (2013). phyloseq: an R package for reproducible interactive analysis and graphics of microbiome census data. PloS one.

[53] Wickham, H. ggplot2: Elegant Graphics for Data Analysis. (2009).

[54] Segata N (2011). Metagenomic biomarker discovery and explanation. Genome biology.

[55] Langille MG (2013). Predictive functional profiling of microbial communities using 16S rRNA marker gene sequences. Nature biotechnology.

[56] Parks DH, Tyson GW, Hugenholtz P, Beiko RG (2014). STAMP: statistical analysis of taxonomic and functional profiles. Bioinformatics.

