# Supplementary material for: Helicobacter pylori-infected C57BL/6 mice with different gastrointestinal microbiota have contrasting gastric pathology, microbial and host immune responses
Source: Sci Rep. 2018 May 22;8:8014. doi: 10.1038/s41598-018-25927-2 (PMC5964229; doi:10.1038/s41598-018-25927-2)
Supplement: Supplementary file 1 — Supplemental information [file 41598_2018_25927_MOESM1_ESM.pdf]

Helicobacter pylori-infected C57BL/6 mice with different gastrointestinal microbiota have contrasting gastric pathology, microbial and host immune responses

Zhongming Ge*, Alex Sheh, Yan Feng, Sureshkumar Muthupalani, Lili Ge, Chuanwu Wang, Susanna Kurnick, Anthony Mannion, Mark T. Whary, James G. Fox*

Supplementary Info

	Log LDA scores		
	**Jax**	**Tac**	**p-value**
	**Stomach**		
Bacteria.Bacteroidetes.Bacteroidia.Bacteroidales.S24_7	4.73		0.014
Bacteria.Actinobacteria.Coriobacteriia.Coriobacteriales.Coriobacteriaceae	3.92		0.027
Bacteria.Actinobacteria.Actinobacteria.Bifidobacteriales.Bifidobacteriaceae	3.79		0.011
Bacteria. Unassigned.Other.Other.Other.Unassigned_Other	3.85		0.011
Bacteria.Firmicutes.Clostridia.Clostridiales.Clostridiaceae	4.04		0.027
Bacteria. Unassigned.Other.Other.Other.Unassigned_Other (Order RF39)	3.70		0.014
Bacteria.Firmicutes.Bacilli.Turicibacterales.Turicibacteraceae	4.71		0.014
Bacteria.Proteobacteria.gammaproteobacteria.Pseudomonadales.Moraxellaceae		4.49	0.029
Bacteria.Bacteroidetes.Bacteroidia.Bacteroidales.Rikenellaceae		3.95	0.029
Bacteria.Proteobacteria.Betaproteobacteria.Burkholderiales.Oxalobacteraceae		3.77	0.029
Bacteria.Deferribacteres.Deferribacteres.Deferribacterales.Deferribacteraceae		3.75	0.029
Bacteria.Bacteroidetes.Bacteroidia.Bacteroidales.Porphyromonadaceae		3.73	0.029
	Colon		
Bacteria.Bacteroidetes.Bacteroidia.Bacteroidales.S24_7	5.12		0.014
Bacteria.Actinobacteria.Actinobacteria.Bifidobacteriales.Bifidobacteriaceae	4.50		0.014
Bacteria. Unassigned.Other.Other.Other.Unassigned_Other	4.20		0.014
Bacteria.Firmicutes.Bacilli.Turicibacterales.Turicibacteraceae	4.16		0.021
Bacteria.Firmicutes.Clostridia.Clostridiales.Lachnospiraceae		4.76	0.043
Bacteria.Deferribacteres.Deferribacteres.Deferribacterales.Deferribacteraceae		4.39	0.014
Bacteria.Bacteroidetes.Bacteroidia.Bacteroidales.Rikenellaceae		4.16	0.014
Bacteria.Verrucomicrobia.Verrucomicrobiae.Verrucomicrobiales.Verrucomicrobiaceae		4.00	0.047
Bacteria.Bacteroidetes.Bacteroidia.Bacteroidales.Porphyromonadaceae		3.99	0.014
	Feces		
Bacteria.Bacteroidetes.Bacteroidia.Bacteroidales.S24_7	5.52		0.034
Bacteria.Actinobacteria.Actinobacteria.Bifidobacteriales.Bifidobacteriaceae	4.60		0.028
Bacteria.Firmicutes.Clostridia.Clostridiales.Ruminococcaceae		5.01	0.034
Bacteria.Bacteroidetes.Bacteroidia.Bacteroidales.Rikenellaceae		4.87	0.019
Bacteria.Bacteroidetes.Bacteroidia.Bacteroidales.Porphyromonadaceae		4.65	0.019
Bacteria.Firmicutes.Clostridia.Clostridiales.Lachnospiraceae		4.62	0.034
Bacteria.Proteobacteria.Gammaproteobacteria.Alteromonadales.Shewanellaceae		4.35	0.029
Bacteria.Firmicutes.Clostridia.Clostridiales.Peptococcaceae		4.13	0.034
Bacteria.Deferribacteres.Deferribacteres.Deferribacterales.Deferribacteraceae		3.84	0.019

Table S2. Gastric, colonic and fecal bacteria (at Family level) affected by *H. pylori* infection

	Log LDA scores					
	Jax			Tac		
	Decreased	Increased	P-value	Decreased	Increased	P-value
	Stomach					
Proteobacteria.Epsilonproteobacteria. Campylobacterales.Helicobacteraceae		3.89	0.004		3.35	0.017
Tenericutes.Mollicutes.Anaeroplasmatales.Anaeroplasmataceae	-3.39		0.042			
Actinobacteria.Actinobacteria.Bifidobacteriales.Bifidobacteriaceae	-3.56		0.017			
Unassigned.Other.Other.Other.Unassigned_Other	-3.48		0.017			
Firmicutes.Clostridia.Clostridiales.Clostridiaceae	-3.90		0.045	-3.44		0.008
Firmicutes.Clostridia.Clostridiales.Peptostreptococcaceae				-3.49		0.022
Firmicutes.Bacilli.Turicibacterales.Turicibacteraceae				-3.85		0.008
	Colon					
Firmicutes.Clostridia.Clostridiales.Dehalobacteriaceae		3.03	0.020			
Actinobacteria.Coriobacteriia.Coriobacteriales.Coriobacteriaceae	-2.89		0.017			
Actinobacteria.Actinobacteria.Bifidobacteriales.Bifidobacteriaceae	-3.00		0.018			
Firmicutes.Bacilli.Lactobacillales.Lactobacillaceae					3.91	0.020
	Feces					
Firmicutes.Clostridia.Clostridiales.Dehalobacteriaceae		3.35	0.028			
Actinobacteria.Actinobacteria.Bifidobacteriales.Bifidobacteriaceae	-3.17		0.014			
Tenericutes.Mollicutes.Anaeroplasmatales.Anaeroplasmataceae	-3.67		0.043			
Firmicutes.Bacilli.Turicibacterales.Turicibacteraceae	-3.75		0.042	-3.80		0.034
Firmicutes.Bacilli.Lactobacillales.Lactobacillaceae					5.10	0.034
Firmicutes.Clostridia.Clostridiales.Mogibacteriaceae					4.84	0.026

Table S3. *In silico* prediction of promoted KEGG pathways in Jax mice (in blue) or Tac mice (in red)

Level_1	Level_2	Level_3	Jax: mean rel. freq. (%)	Jax: std. dev. (%)	Tac: mean rel. freq. (%)	Tac: std. dev. (%)	p-values	p-values (corrected)
**Metabolism**	**Amino Acid Metabolism**	**Tyrosine metabolism**	**0.32469**	**0.02621**	**0.44051**	**0.07407**	**0.00015**	**0.00329**
**Metabolism**	**Amino Acid metabolism**	**Amino acid metabolism_Unclassified**	**0.20091**	**0.01241**	**0.18591**	**0.00567**	**0.00239**	**0.01505**
**Metabolism**	**Amino Acid Metabolism**	**Amino acid related enzymes**	**1.41563**	**0.04287**	**1.35254**	**0.01971**	**0.00041**	**0.00588**
**Metabolism**	**Amino Acid Metabolism**	**Phenylalanine, tyrosine and tryptophan biosynthesis**	**0.66696**	**0.14487**	**0.47503**	**0.13294**	**0.00582**	**0.02650**
**Metabolism**	**Biosynthesis of Other Secondary Metabolites**	**Isoquinoline alkaloid biosynthesis**	**0.03404**	**0.00713**	**0.02201**	**0.00702**	**0.00112**	**0.01017**
**Metabolism**	**Biosynthesis of Other Secondary Metabolites**	**Novobiocin biosynthesis**	**0.09762**	**0.02307**	**0.06572**	**0.02101**	**0.00417**	**0.02173**
**Metabolism**	**Biosynthesis of Other Secondary Metabolites**	**Phenylpropanoid biosynthesis**	**0.15779**	**0.04242**	**0.102**	**0.03484**	**0.00435**	**0.02231**
**Metabolism**	**Biosynthesis of Other Secondary Metabolites**	**Tropane, piperidine and pyridine alkaloid biosynthesis**	**0.077**	**0.01566**	**0.05614**	**0.01629**	**0.00847**	**0.03386**
**Human Diseases**	**Cancers**	**Prostate cancer**	**0.03292**	**0.00831**	**0.02068**	**0.00759**	**0.00260**	**0.01551**
**Metabolism**	**Carbohydrate Metabolism**	**Butanoate metabolism***	**0.76601**	**0.08248**	**0.91239**	**0.06686**	**0.00030**	**0.00524**
**Metabolism**	**Carbohydrate Metabolism**	**Inositol phosphate metabolism**	**0.15937**	**0.02787**	**0.24508**	**0.03641**	**0.00001**	**0.00096**
**Metabolism**	**Carbohydrate Metabolism**	**Pentose phosphate pathway**	**0.80784**	**0.03991**	**0.86313**	**0.04202**	**0.00681**	**0.02976**
**Metabolism**	**Carbohydrate Metabolism**	**Propanoate metabolism***	**0.59454**	**0.09169**	**0.76324**	**0.08258**	**0.00033**	**0.00541**
**Metabolism**	**Carbohydrate Metabolism**	**Pyruvate metabolism***	**1.02851**	**0.01937**	**1.2075**	**0.09359**	**0.00001**	**0.00141**
**Metabolism**	**Carbohydrate Metabolism**	**Amino sugar and nucleotide sugar metabolism#**	**1.36384**	**0.09068**	**1.24187**	**0.08083**	**0.00476**	**0.02330**
**Metabolism**	**Carbohydrate Metabolism**	**Fructose and mannose metabolism#**	**0.83923**	**0.08241**	**0.73688**	**0.07271**	**0.00801**	**0.03242**
**Metabolism**	**Carbohydrate Metabolism**	**Galactose metabolism#**	**0.80594**	**0.09625**	**0.69488**	**0.08224**	**0.01170**	**0.04412**
**Metabolism**	**Carbohydrate Metabolism**	**Starch and sucrose metabolism#**	**0.91916**	**0.10278**	**0.78816**	**0.08256**	**0.00513**	**0.02472**
**Cellular Processes and Signaling**	**Cell division**	**Cell division_Unclassified**	**0.06062**	**0.01573**	**0.03537**	**0.00794**	**0.00020**	**0.00391**
**Cellular Processes**	**Cell Growth and Death**	**Apoptosis**	**0.00153**	**0.00156**	**0.01449**	**0.00793**	**0.00006**	**0.00212**
**Organismal Systems**	**Digestive System**	**Protein digestion and absorption**	**0.00839**	**0.0055**	**0.00202**	**0.00083**	**0.00170**	**0.01294**

Organismal Systems	Endocrine System	Adipocytokine signaling pathway	0.03852	0.01236	0.02368	0.00559	0.00249	0.01512
Organismal Systems	Endocrine System	Insulin signaling pathway#	0.054	0.01801	0.03361	0.01498	0.01225	0.04564
Organismal Systems	Endocrine System	Progesterone-mediated oocyte maturation	0.03291	0.00832	0.02043	0.00732	0.00196	0.01338
Metabolism	Energy Metabolism	Carbon fixation in photosynthetic organisms	0.56654	0.03796	0.52247	0.02406	0.00563	0.02676
Metabolism	Energy metabolism	Energy metabolism_Unclassified	0.83812	0.09597	0.66251	0.10186	0.00076	0.00829
Metabolism	Energy Metabolism	Methane metabolism	1.20874	0.07995	1.11594	0.0584	0.00767	0.03186
Metabolism	Energy Metabolism	Oxidative phosphorylation	1.06587	0.08631	0.96249	0.03482	0.00219	0.01438
Organismal Systems	Excretory System	Proximal tubule bicarbonate reclamation	0.02603	0.00813	0.04292	0.01607	0.00763	0.03207
Genetic Information Processing	Folding, Sorting and Degradation	Sulfur relay system	0.2534	0.05587	0.36061	0.05766	0.00042	0.00572
Genetic Information Processing	Folding, Sorting and Degradation	Ubiquitin system	0.00641	0.00457	0.02121	0.00808	0.00006	0.00206
Genetic Information Processing	Folding, Sorting and Degradation	Proteasome	0.03337	0.00838	0.02048	0.00729	0.00153	0.01193
Genetic Information Processing	Folding, Sorting and Degradation	Protein export	0.5698	0.03005	0.52668	0.02638	0.00278	0.01627
Genetic Information Processing	Folding, Sorting and Degradation	Protein processing in endoplasmic reticulum	0.05872	0.00714	0.04786	0.00526	0.00095	0.00943
Cellular Processes and Signaling	Germination	Germination_Unclassified	0.05126	0.0249	0.01709	0.01416	0.00120	0.01060
Metabolism	Glycan Biosynthesis and Metabolism	Glycosaminoglycan degradation#	0.06854	0.03994	0.01885	0.01234	0.00124	0.01066
Metabolism	Glycan Biosynthesis and Metabolism	Glycosphingolipid biosynthesis - ganglio series#	0.04443	0.03073	0.00841	0.00436	0.00152	0.01217
Metabolism	Glycan Biosynthesis and Metabolism	Glycosphingolipid biosynthesis - globo series#	0.12934	0.05382	0.06733	0.02478	0.00350	0.01913
Metabolism	Glycan Biosynthesis and Metabolism	N-Glycan biosynthesis#	0.01782	0.00837	0.00909	0.00395	0.00734	0.03166
Metabolism	Glycan Biosynthesis and Metabolism	Other glycan degradation	0.32593	0.14732	0.17931	0.08498	0.01301	0.04688
Organismal Systems	Immune System	Antigen processing and presentation	0.03291	0.00832	0.02043	0.00732	0.00196	0.01366

Organismal Systems	Immune System	NOD-like receptor signaling pathway	0.03293	0.00831	0.02043	0.00732	0.00193	0.01379
Human Diseases	Infectious Diseases	Staphylococcus aureus infection	0.04207	0.04111	0.10005	0.05042	0.01062	0.04097
Human Diseases	Infectious Diseases	Epithelial cell signaling in Helicobacter pylori infection	0.07095	0.01712	0.04587	0.01757	0.00416	0.02202
Metabolism	Lipid Metabolism	Biosynthesis of unsaturated fatty acids*	0.13432	0.03082	0.17545	0.0206	0.00221	0.01420
Metabolism	Lipid Metabolism	Fatty acid biosynthesis*	0.37939	0.02781	0.50576	0.06514	0.00002	0.00105
Metabolism	Lipid Metabolism	Fatty acid metabolism*	0.30793	0.07371	0.3885	0.05053	0.00987	0.03901
Metabolism	Lipid Metabolism	Lipid biosynthesis proteins*	0.54039	0.02428	0.59328	0.03142	0.00043	0.00562
Metabolism	Lipid Metabolism	Primary bile acid biosynthesis*	0.04784	0.01348	0.07433	0.02357	0.00583	0.02621
Metabolism	Lipid Metabolism	Secondary bile acid biosynthesis*	0.04771	0.01341	0.07408	0.02345	0.00581	0.02684
Metabolism	Metabolism of cofactors and vitamins	Metabolism of cofactors and vitamins_Unclassified	0.13037	0.04602	0.2097	0.04547	0.00094	0.00960
Metabolism	Metabolism of Cofactors and Vitamins	Retinol metabolism	0.03666	0.01012	0.07369	0.02071	0.00006	0.00235
Metabolism	Metabolism of Cofactors and Vitamins	Thiamine metabolism	0.45687	0.02038	0.5145	0.04672	0.00189	0.01379
Metabolism	Metabolism of Cofactors and Vitamins	Ubiquinone and other terpenoid-quinone biosynthesis	0.2328	0.06355	0.3343	0.08802	0.00780	0.03200
Metabolism	Metabolism of Other Amino Acids	Selenocompound metabolism	0.3454	0.01118	0.3737	0.01157	0.00002	0.00105
Metabolism	Metabolism of Other Amino Acids	Cyanoamino acid metabolism	0.30343	0.04007	0.22576	0.03239	0.00012	0.00321
Metabolism	Metabolism of Terpenoids and Polyketides	Biosynthesis of siderophore group nonribosomal peptides	0.02495	0.00831	0.04907	0.011	0.00002	0.00096
Metabolism	Metabolism of Terpenoids and Polyketides	Tetracycline biosynthesis	0.09723	0.00964	0.16971	0.03778	0.00001	0.00077
Metabolism	Metabolism of Terpenoids and Polyketides	Biosynthesis of vancomycin group antibiotics	0.05583	0.00859	0.04634	0.00284	0.00344	0.01911

Legends for supplemental Figures:

Figure S1. Gastric histologic activity index (GHAI). GHAI is an aggregate of scores on an ascending scale from 0 to 4 for inflammation, epithelial defects, atrophy, hyperplasia, pseudopyloric metaplasia, dysplasia, hyalinosis, and mucous metaplasia. Infected mice developed more severe gastric pathology compared to sham controls. There is no significant difference between infected Jax and Tac B6 females.

Figure S2. mRNA levels of the target genes in ileum. All the mRNA levels were normalized to the expression of the housekeeping gene *Gapdh*. The *y* axes represent the mean fold changes (± standard deviations) of the mRNA levels in reference to uninfected Jax controls. P values: * ***<0.001, ****<0.0001.

Figure S3. Gastrointestinal microbial composition related to **Figure 5** and **Figure 6**. Mean relative abundance of bacterial taxa (>0.1%) at Plylum (A) and Family levels in gastric, colonic and fecal samples from PMSS1-infected and control Jax and Tac B6 female mice was presented.

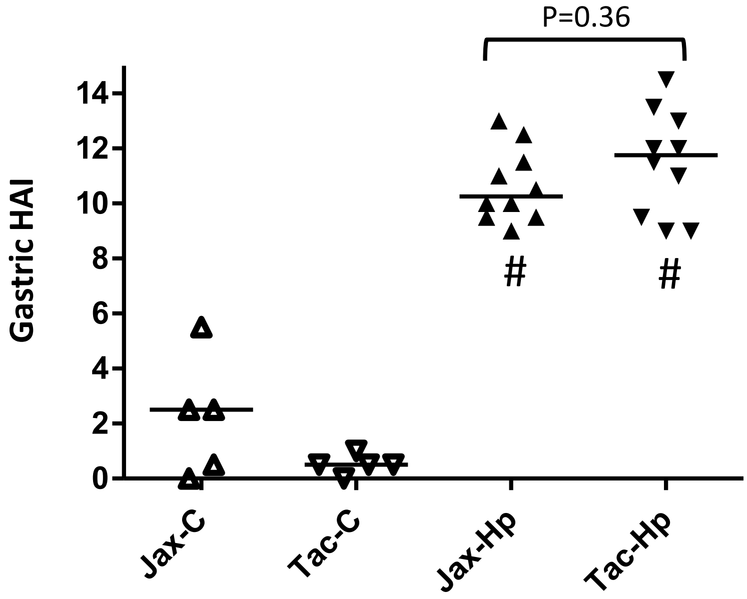

Figure S1

Transcript levels of ileal genes

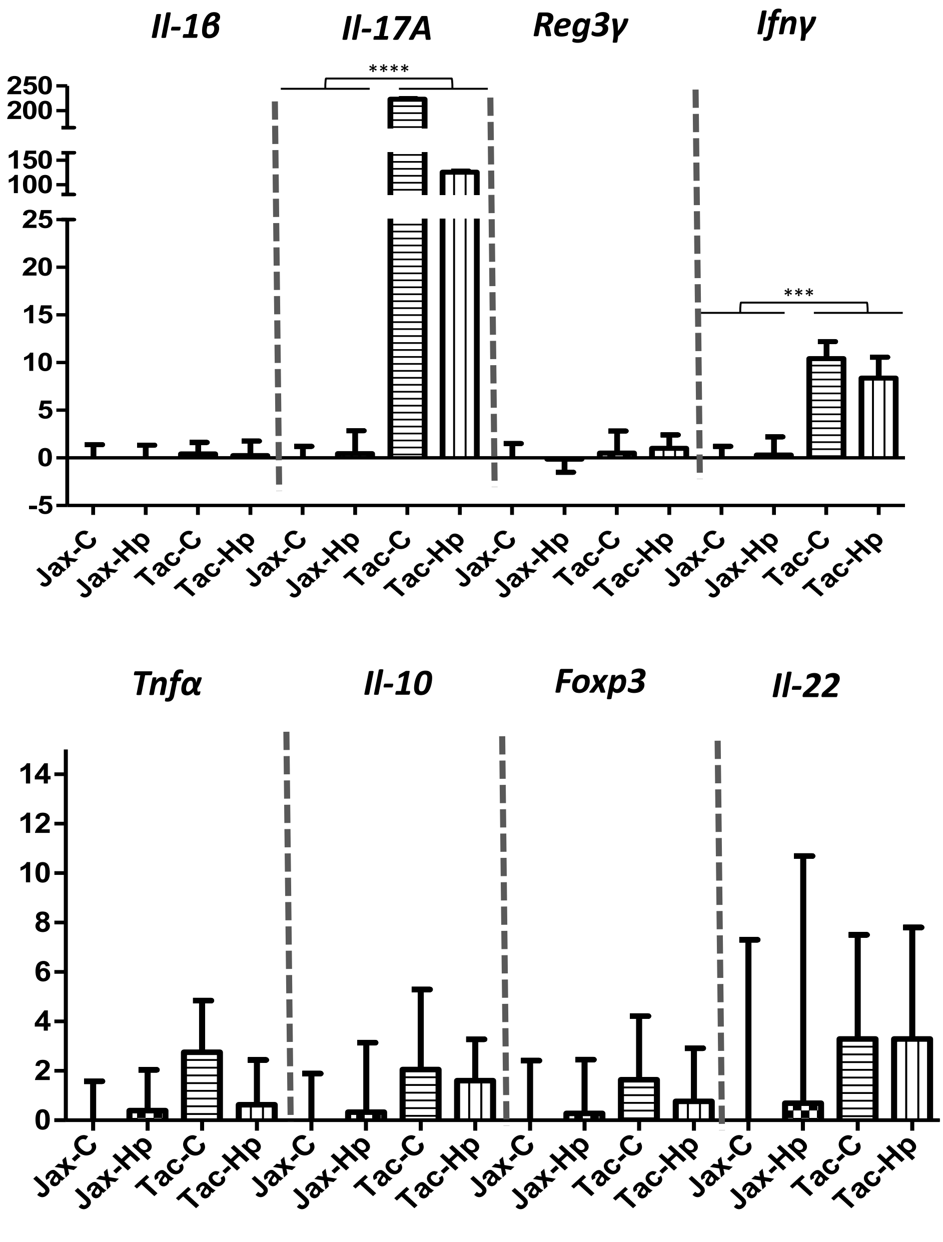

Figure S2

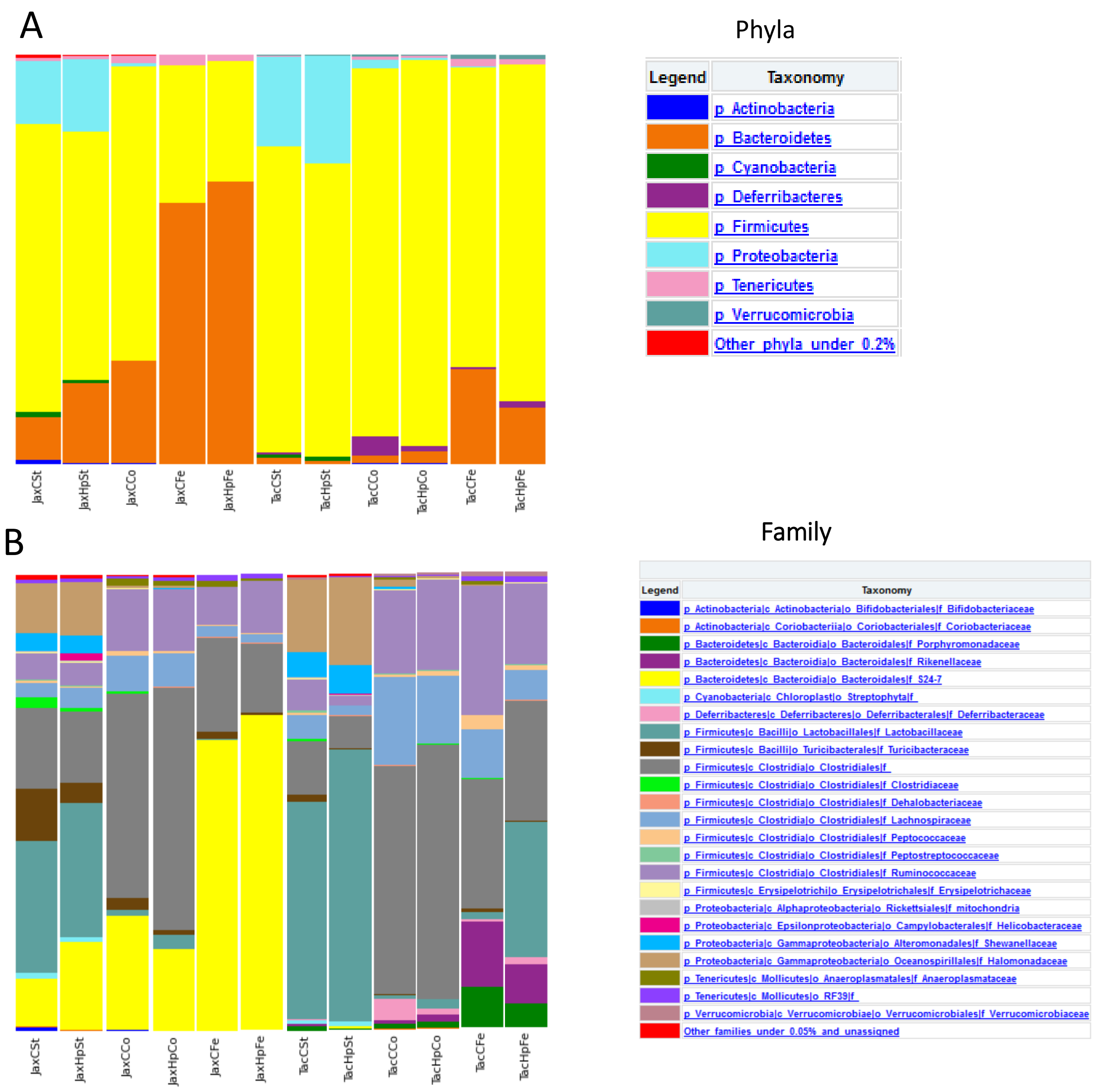
A
Phyla
Legend
Taxonomy
p Actinobacteria
p Bacteroidetes
p Cyanobacteria
p Deferribacteres
p Firmicutes
p Proteobacteria
p Tenericutes
p Verrucomicrobia
Other phyla under 0.2%
JaxCSt
JaxHpSt
JaxCCo
JaxCFe
JaxHpFe
TacCSt
TacHpSt
TacCCo
TacHpCo
TacCFe
TacHpFe
B
Family
Legend
Taxonomy
p Actinobacteria|c Actinobacteria|o Bifidobacteriales|f Bifidobacteriaceae
p Actinobacteria|c Coriobacteriia|o Coriobacteriales|f Coriobacteriaceae
p Bacteroidetes|c Bacteroidia|o Bacteroidales|f Porphyromonadaceae
p Bacteroidetes|c Bacteroidia|o Bacteroidales|f Rikenellaceae
p Bacteroidetes|c Bacteroidia|o Bacteroidales|f S24-7
p Cyanobacteria|c Chloroplast|o Streptophyta|f
p Deferribacteres|c Deferribacteres|o Deferribacterales|f Deferribacteraceae
p Firmicutes|c Bacilli|o Lactobacillales|f Lactobacillaceae
p Firmicutes|c Bacilli|o Turicibacterales|f Turicibacteraceae
p Firmicutes|c Clostridia|o Clostridiales|f
p Firmicutes|c Clostridia|o Clostridiales|f Clostridiaceae
p Firmicutes|c Clostridia|o Clostridiales|f Dehalobacteriaceae
p Firmicutes|c Clostridia|o Clostridiales|f Lachnospiraceae
p Firmicutes|c Clostridia|o Clostridiales|f Peptococcaceae
p Firmicutes|c Clostridia|o Clostridiales|f Peptostreptococcaceae
p Firmicutes|c Clostridia|o Clostridiales|f Ruminococcaceae
p Firmicutes|c Erysipelotrichi|o Erysipelotrichales|f Erysipelotrichaceae
p Proteobacteria|c Alphaproteobacteria|o Rickettsiales|f mitochondria
p Proteobacteria|c Epsilonproteobacteria|o Campylobacterales|f Helicobacteraceae
p Proteobacteria|c Gammaproteobacteria|o Alteromonadales|f Shewanellaceae
p Proteobacteria|c Gammaproteobacteria|o Oceanospirillales|f Halomonadaceae
p Tenericutes|c Mollicutes|o Anaeroplasmatales|f Anaeroplasmataceae
p Tenericutes|c Mollicutes|o RF39|f
p Verrucomicrobia|c Verrucomicrobiae|o Verrucomicrobiales|f Verrucomicrobiaceae
Other families under 0.05% and unassigned
JaxCSt
JaxHpSt
JaxCCo
JaxHpCo
JaxCFe
JaxHpFe
TacCSt
TacHpSt
TacCCo
TacHpCo
TacCFe
TacHpFe

Figure S3
